# *De novo* transcriptomic assembly and mRNA expression patterns of *Botryosphaeria dothidea* infection with mycoviruses chrysovirus 1 (BdCV1) and partitivirus 1 (BdPV1)

**DOI:** 10.1186/s12985-018-1033-4

**Published:** 2018-08-13

**Authors:** Lihua Wang, Hui Luo, Wangcheng Hu, Yuekun Yang, Ni Hong, Guoping Wang, Aiming Wang, Liping Wang

**Affiliations:** 1 State Key Laboratory of Agricultural Microbiology, Wuhan, Hubei 430070 People’s Republic of China; 2 College of Plant Science and Technology, Huazhong Agricultural University, Wuhan, Hubei 430070 People’s Republic of China; 3 Key Lab of Plant Pathology of Hubei Province, Wuhan, Hubei 430070 People’s Republic of China; 4 London Research and Development Centre, Agriculture and Agri-Food Canada, London, ON N5V 4T3 Canada

**Keywords:** Pear ring rot disease, *Botryosphaeria dothidea*, *De novo* transcriptional sequencing and analysis, Mycovirus, Chrysovirus, KEGG pathway, GO enrichment analysis, Differential expression genes (DEGs), Fungi-mycovirus interaction

## Abstract

**Background:**

Pear ring rot, caused by *Botryosphaeria* species, is responsible for substantial economic losses by causing severe recession of pear tree growth in China. Mycovirus-mediated hypovirulence in plant pathogenic fungi is a crucial biological method to control fungal diseases.

**Methods:**

We conducted a large-scale and comprehensive transcriptome analysis to identify mRNA in *B. dothidea* in response to mycovirus. *De novo* sequencing technology from four constructed libraries of LW-C (Botryosphaeria dothidea chrysovirus 1, BdCV1), LW-P (Botryosphaeria dothidea partitivirus 1, BdPV1), LW-CP (LW-1 strain infection with BdCV1 and BdPV1), and Mock (free virus) was used to investigate and compare gene expression changes in *B.dothidea* strains infected with mycovirus.

**Results:**

In total, 30,058 Unigenes with an average length of 2128 bp were obtained from 4 libraries of *B. dothidea* strains. These were annotated to specify their classified function. We demonstrate that mRNAs of *B. dothidea* strains in response to mycovirus are differentially expressed. In total, 5598 genes were up-regulated and 3298 were down-regulated in the LW-CP group, 4468 were up-regulated and 4291 down-regulated in the LW-C group, and 2590 were up-regulated and 2325 down-regulated in the LW-P group. RT-qPCR was used to validate the expression of 9 selected genes. The *B. dothidea* transcriptome was more affected by BdCV1 infection than BdPV1. We conducted GO enrichment analysis to characterize gene functions regulated by *B. dothidea* with mycovirus infection. These involved metabolic process, cellular process, catalytic activity, transporter activity, signaling, and other biological pathways. KEGG function analysis demonstrated that the enriched differentially expressed genes are involved in metabolism, transcription, signal transduction, and ABC transport. mRNA is therefore involved in the interaction between fungi and mycovirus. In addition, changes in differential accumulation levels of *cp* and *RdRp* of BdCV1 and BdPV1 in *B. dothidea* strains were evaluated, revealing that the accumulation of BdCV1 and BdPV1 is related to the phenotype and virulence of *B. dothidea* strain LW-1.

**Conclusions:**

The identification and analysis of mRNAs from *B. dothidea* was first reported at the transcriptome level. Our analysis provides further insight into the interaction of *B. dothidea* strains infection with chrysovirus 1 (BdCV1) and partitivirus 1 (BdPV1) at the transcriptome level.

**Electronic supplementary material:**

The online version of this article (10.1186/s12985-018-1033-4) contains supplementary material, which is available to authorized users.

## Background

Pear ring rot, caused by the destructive pathogen *Botryosphaeria*, is responsible for substantial economic losses through widespread fruit rots and stem canker. This caused a severe recession in the growth of pear fruit trees in China [1–5]. Due to the lack of disease-resistant varieties, many fungicides are recommended for treatment of Botryosphaeria canker, which induces drug resistance and environmental pollution [3]. It is urgent to find new, safe, and effective means to control pear ring rot disease. Mycovirus-mediated hypovirulence in plant pathogenic fungi is a powerful method to control fungal crop diseases such as the hypovirulent strain of *C. parasitica* (CHV1) to heal cankers of chestnut blight, and Sclerotinia sclerotiorum hypovirulence-associated DNA virus 1 (SsHADV-1) to control the rapeseed stem rot [6–13]. Recent studies of fruit tree fungal diseases have reported that the mycovirus Rosellinia necatrix megabirnavirus 1 (RnMRV1) has potential to control white root rot (*Rosellinia necatrix*) diseases of fruit trees [8]. In recent years, several dsRNA mycoviruses have been identified and sequenced in *B. dothidea* strains isolated from pear trees with ring rot and stem wart symptoms in China [14, 15]. Chrysovirus BdCV1 and partitivirus BdPV1 mixed infection results in hypovirulent *B. dothidea* LW-1 strain in our study, revealing BdCV1 to be the first mycovirus found to be responsible for hypovirulence (reduced virulence), growth rate, and phenotypic sectorization of the phytopathogenic fungus *B. dothidea* [16]. BdCV1 is therefore a good candidate for biological control of apple and pear ring spot diseases, while BdPV1 does not cause any visible symptoms in virulent pathogenicity [5, 16].

High throughput sequencing technology provided comprehensive information on gene expression. Measuring the expression patterns of mRNAs at the transcriptome level from fungi infection with mycovirus is vital to reveal the mechanism of mycovirus-mediated hypovirulence in pathogenic fungi [6, 17, 18]. Only a limited number of studies, using high throughput sequencing technologies and bioinformatics, have demonstrated transcriptional or translational changes in fungi infection with mycovirus, such as *Cryphonectria parasitica*, *Aspergillus fumigatus*, *Neurospora crassa*, *Sclerotinia sclerotiorum,* and *Fusarium graminearum* [17–24]. For instance, 150 genes representing a wide spectrum of biological functions were down-regulated in the strain Ep-1PN by Sclerotinia Sclerotiorum Debilitation-associated RNA Virus (SsDRV), of which *S. sclerotiorum* integrin like gene (*SSITL*) was verified affect pathogenic fungus virulence and growth rate [20, 23]. Genes associated with viral replication, maintenance of viral life cycle, transcription and translation machinery, and signal transduction were up-regulated while those including membrane-associated transporters and cellular transport systems were down-regulated based on a genome-wide transcriptome analysis of *F. graminearum* infected with Fusarium graminearum virus 1 (FgV1) [18, 21, 22, 25].

The main objective of our study is to characterize the mRNAs from *B. dothidea* involved in biological processes associated with the host infecting with mycovirus. Currently, there are no studies published on the *B. dothidea* transcriptome. No direct and detailed functional genomics resources in public databases for understanding the molecular mechanism of *B. dothidea* exist except the draft genome sequences from two *B. dothidea* strains reported, the pathogen of Apple ring rot disease and isolated from grapevine host, respectively [26, 27]. Therefore, large-scale transcriptome sequencing using Illumina sequencing technology was performed to explore the potential mRNAs expression in *B. dothidea* related to pathogenic factors and hypovirulence determinants in response to two mycoviruses BdCV1 and BdPV1.

## Materials 

### *Botryosphaeria dothidea* strains and infection with BdCV1 and BdPV1

Three *B. dothidea* strains, LW-1, LW-C, and LW-P, infected with hypovirulent and non-hypovirulent mycovirus were generated [5, 16]. LW-1 (or designated as LW-CP) was infected with Botryosphaeria dothidea chrysovirus 1 (BdCV1) and Botryosphaeria dothidea partitivirus 1 (BdPV1), which was isolated and identified from the trunks of sand pear‘Jinshuiyihao’ cv., collected in Wuhan city, Hubei province of China [5]. The LW-P strain infection with BdPV1 and LW-C with BdCV1 were derived from LW-1 by single hyphae isolation, respectively, as previously described [5, 16]. Meanwhile, mock derived from LW-C strain by protoplast isolation was virus-free as negative control. The fungal strains were grown at 25 °C in darkness on potato dextrose agar (PDA) medium and stored on PDA plate at − 4 °C or in glycerin at − 80 °C.

### Total RNA extraction from *B. dothidea* strains

The mycelia of the mycovirus-infected three strains and the virus-free strain of *B. dothidea* were harvested on 5 day of PDA culture. The mycelia were frozen and ground using liquid nitrogen for nucleic acid extraction. Total RNAs were isolated and obtained with TRIzol Reagent (Invitrogen, USA), phenol and chloroform to remove proteins and ethanol precipitation according to manufacturer’s instructions. Total RNAs digested by DNase I (Takara, Dalian, China) were precipitated with ethanol and dissolved in DEPC treated water to be used as template for Next Genomics Sequencing (NGS). The RNA integrity Number (RIN > 7) and concentration of obtained tRNAs were further assessed using the Bioanalyzer Agilent 2100 and NanoDrop spectrophotometer (Agilent, USA). High quality samples were used to construct the four *de novo* libraries.

### cDNA library construction and *de novo* sequencing

For each RNA-seq library, total RNA from three biological repetitions were pooled. Following the total RNA extraction and treatment with DNase I, mRNA was isolated and enriched by Oligo (dT). mRNA was cut into small fragments and cDNA synthesis was performed with random hexamer primers. PCR amplification was then prepared for library construction according to the Illumina RNA library protocol for transcriptome sequencing. Then, the quantification of the four cDNA libraries was verified by the Agilent 2100 Bioanalyzer and ABI StepOnePlus Real-Time PCR System. Finally, the libraries were sequenced using the HiSeq 4000 instrument (Illumina, USA) at the Beijing Genomics Institute (BGI) (Shenzhen, Guangdong province, China).

### Transcriptome data processing, *de novo* assembly, and functional annotation

Internal software was used to filter raw reads to generate clean reads data via the following processes: 1) The removal of adaptor-polluted reads, 2) removal of reads containing a high content (> 5%) of unknown bases (N), 3) removal of low-quality reads. Following filtering, the remaining reads were used for downstream analyses. Trinity was used to perform *de novo* assembly with clean reads which PCR duplication removed, and Tgicl was used to cluster transcripts to Unigenes. The sequence by Trinity was called transcripts. Then, gene family clustering with Tgicl for each sample’s Unigene to obtain final Unigenes was performed for downstream analyses. The Unigenes were divided into two classes, including clusters and singletons. The prefix used for clusters was CL with the cluster id behind it (in one cluster, there are several Unigenes with more than 70% similarity between them). The prefix used for singletons was Unigene. For unigene functional annotation analysis, NT (ftp://ftp.ncbi.nlm.nih.gov/blast/db), NR (ftp://ftp.ncbi.nlm.nih.gov/blast/db), GO (http://geneontology.org), COG (http://www.ncbi.nlm.nih.gov/COG), KEGG (http://www.genome.jp/kegg), SwissProt (http://ftp.ebi.ac.uk/pub/databases/swissprot), and InterPro (http://www.ebi.ac.uk/interpro) were used as functional databases. Blasts aligned unigenes to NT (NCBI non-redundant nucleotide sequences), NR (NCBI non-redundant protein sequences), COG (Cluster of Orthologous Groups), KEGG (Kyoto Encyclopedia of Genes and Genome), and SwissProt to obtain the annotation. Blast2GO with NR annotation to obtain the GO (Gene Ontology) annotation, and InterProScan5 was used to get the InterPro annotation [28–30]. The functional databases with a priority order of NR, SwissProt, KEGG, and COG were selected to determine the sequence annotation of unigenes. Coding sequences (CDS) were extracted from sequences of unigenes that best mapped to functional databases. ESTScan was used to predict coding regions of unigenes if no hits were obtained in any database mentioned above [31].

### Differential gene expression analysis of mRNA in response to mycovirus

*De novo* sequencing from four constructed cDNA libraries designated as LW-CP, LW-C, LW-P, and Mock was performed. To analyze the differentially expressed genes (DEGs) between two samples in response to mycovirus, clean reads to assembled unigenes using Bowtie2 were mapped [32]. Then, gene expression value was calculated with RSEM [33]. DEGs were identified with PossionDis based on the Poisson distribution, which was performed as described by Audic et al. [34]. The parameters of PossionDis was set as |Fold Change | > 2.0-fold (|log_2_FC| > 1) with a false discovery rate (FDR) < 0.001.

### Clustering analysis of DEGs of *B. dothidea* strains infection with mycovirus

With DEGs, pheatmaps of hierarchical clustering were performed using the heatmap.2 package in R software [35]. For clustering more than two groups, we used the intersecting DEGs.

### GO and KEGG pathway analysis of DEGs

Gene Ontology (GO) classification and functional enrichment was performed on DEGs. GO displayed three ontologies including molecular function, cellular component, and biological process. In addition, KEGG pathway functional enrichment was also analyzed using a corrected *P*-value less than 0.05 for significantly enriched DEGs.

### RT-qPCR analysis of gene mRNA expression level

RT-qPCR was used to validate DEG expression levels obtained from sequencing and to assess the expression levels of *cp* and *RdRp* from BdCV1 and BdPV1. Primers used are listed in Additional file 8. Total RNA was extracted from the four groups: Mock, LW-CP, LW-C, and LW-P cultured for 5 d, 10 d and 15 d using TRIzol (Invitrogen, USA) according to manufacturer’s instructions. Total RNA was digested by DNase I and used as template (Takara, Dalian, China). The cDNA was obtained to perform reverse transcription using PrimeScript™ RT reagent Kit with genomic DNA Eraser (Takara, Dalian, China). According to the manufacturer’s instruction, qPCR was performed using 2.5 μl diluted cDNA, 10 μl of the SYBR *Premix Ex Taq* II PCR mixture (Tli RNaseH Plus) (Takara, Dalian, China), 1 μl of each 5 mM primer, and deionized water to a final volume 20 μl. All reactions were run in triplicate. The qPCR was performed on the Bio-Rad iQTM5 Real-time System machine (BIO-RAD, USA). The products were verified by melt curve analysis. The *18 s* gene from *B.dothidea* strains was used as the internal reference for standardization of cDNA expression levels from samples. mRNA relative expression level changes were quantified by a comparative *C*_T_ method (ΔΔC_T_) using the formula 2^-ΔΔCt^ [36].

## Results

### Differential accumulation of *cp* and *RdRp* in *B. dothidea* infection with BdCV1 and BdPV1

**Fig. 1:**
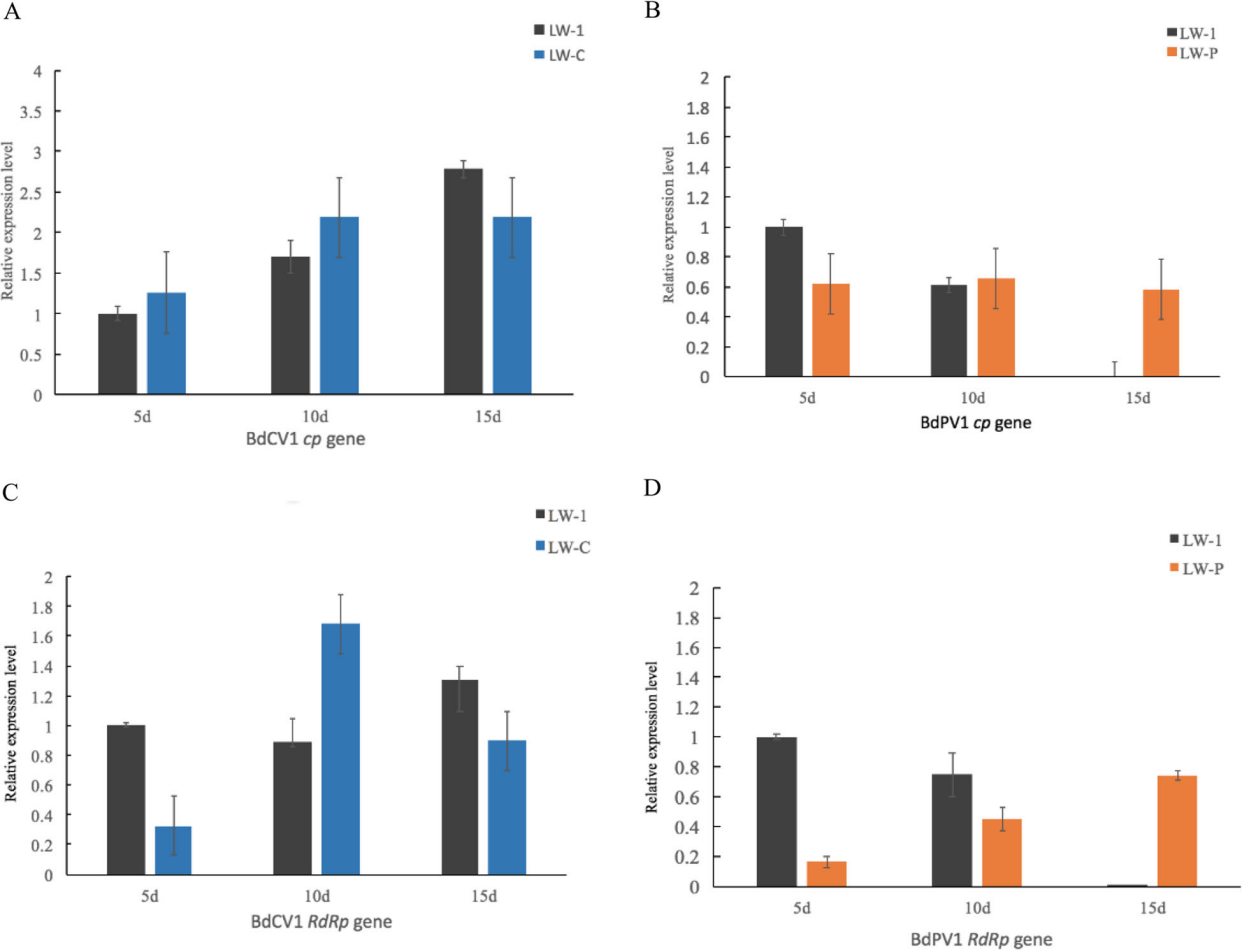
The expression analysis of *cp* and *RdRp* from single or mixed mycovirus chrysovirus 1(BdCV1) and parititvirus 1 (BdPV1) infection of *B. dothidea* strains cultured at 5 d, 10 d, and 15 d by RT-qPCR, respectively

To determine the culture time point to analyze the *B. dothidea* transcriptome in response to mycovirus infection, the relative expression levels of *cp* and *RdRp* mRNA from BdCV1 and BdPV1 in LW-1, LW-C, and LW-P cultured for 5 d, 10 d, and 15 d were quantified by RT-qPCR analysis. The results demonstrate that the relative expression levels of *cp* and *RdRp* mRNA from BdCV1 and BdPV1 are expressed differentially in *B. dothidea* strains LW-C, LW-P, and LW-1 infected with single and mixed mycovirus. In general, BdCV1 viral accumulation increased, while BdPV1 viral accumulation decreased slowly in LW-1 strain cultured periods from 5 to 15 d (Fig. 1).

### *De novo* sequencing and unigene assembly of *B. dothidea* strains

**Fig. 2:**
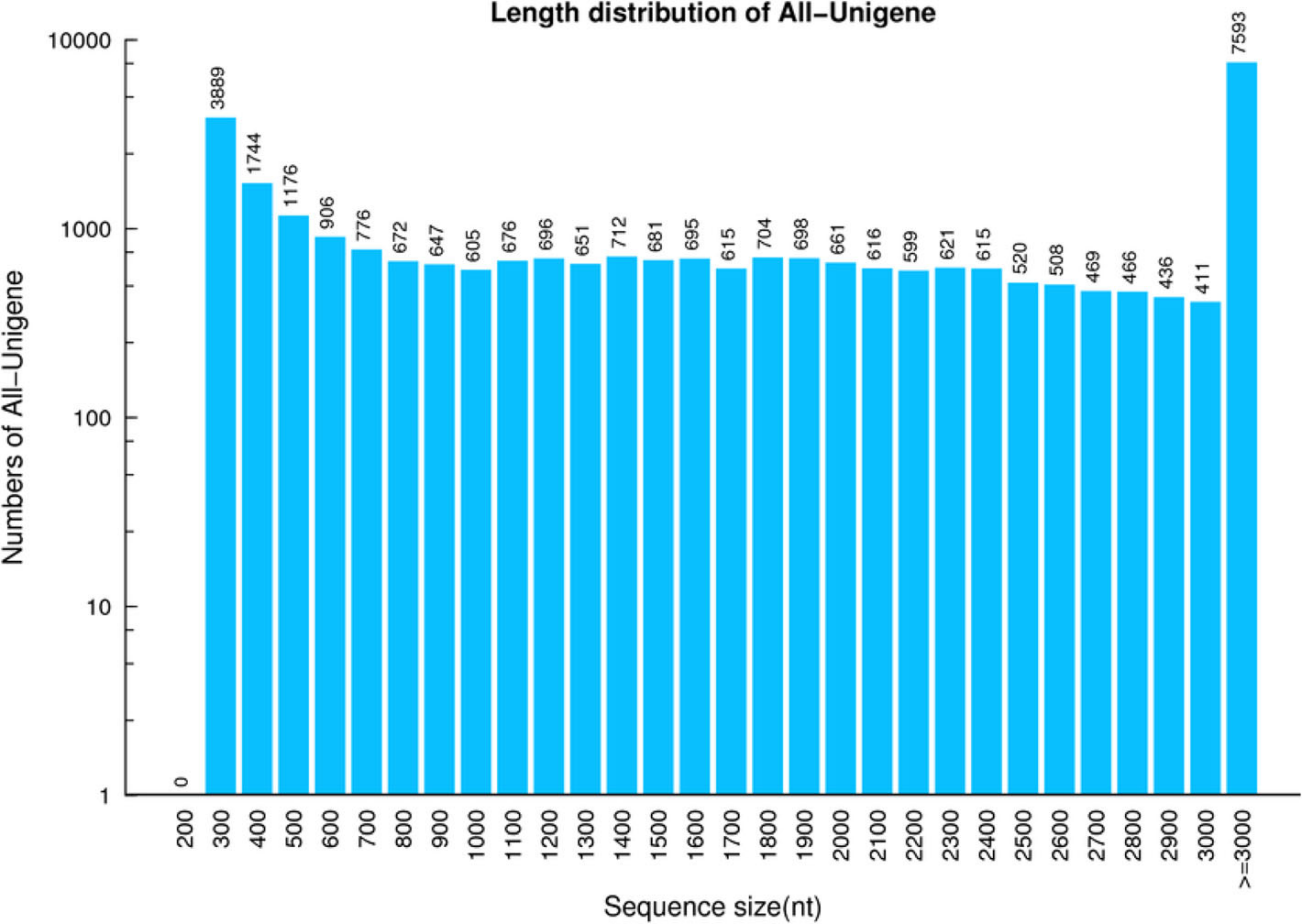
The length distribution of assembled *B. dothidea* unigenes from four constructed libraries by *de novo* sequencing. X axis indicates the length of unigenes. Y axis indicates the number of unigenes

The mycelia of the mycovirus-infected three strains and the virus-free strain of *B. dothidea* at 5 d grown on PDA were collected for RNA extraction to be further used for *De novo* sequencing. The *B. dothidea* transcriptome was sequenced and assembled *de novo*. This is a valuable resource since there are no current genomic data available for *B. dothidea* except two *B.dothidea* strains reported [26, 27]. Four libraries designated as LW-CP, LW-C, LW-P, and Mock were constructed to conduct the comprehensive genome-wide transcriptome analysis following Illumina Hiseq 4000 platform sequencing. In total, clean reads [47.65 Mb (98.4% of raw reads)] corresponding of 4.77 Gb bases from LW-C, 4.67 Gb bases from LW-CP [46.71 Mb (98.38%)], 4.75 Gb bases from LW-P [47.53 Mb (98.24%)], and 4.68 Gb bases from Mock [46.84 Mb (98.16%)] were obtained after trimming, and then assembled using *de novo* assembly program of Trinity (Additional file 1: Table S1). The assembly of the clean reads resulted in 26,461 transcripts for LW-C, 30,707 for LW-CP, 25,808 for LW-P, and 25,298 for Mock. Tgicl was used to cluster transcripts to unigenes. The clustering quality metrics are shown, including of 23,056 unigenes for LW-C, 27,554 for LW-CP, 22,403 for LW-P, and 22,068 for Mock, which reveals the effect of mycovirus infection on a number of gene expression at the transcriptome level. In total, 30,058 unigenes with an average length of 2128 bp and N50 length of 3338 bp were obtained from 4 libraries of *B. dothidea* strains (Additional file 2: Table S2). The size distribution of these unigenes ranged from 300 bp with 3889 unigenes to higher than 3000 bp with 7593 unigenes as shown in Fig. 2. To validate the unigene sequence by *de novo* assembly, RT-PCR and sequencing were used to measure seven unigenes selected randomly. Results demonstrated that they were more than 98% identity comparable to *de novo* sequencing (Additional file 3: Table S3). This is the first report of *B. dothidea* transcriptome.

### Functional annotation of predicted proteins from *B. dothidea* unigenes

Thirty thousand fifty eight non-redundant sequences were submitted to a BLASTx search and annotated to classify function from seven public functional databases including NR, NT, GO, COG, KEGG, Swissprot, and Interpro. In total, the number of unigenes annotated to least one functional database was 24,836 (82.63%). 24,024 (NR: 79.93%), 13,501 (NT: 44.92%), 18,859 (Swissprot: 62.74%), 15,159 (COG: 50.43%), 19,722 (KEGG: 65.61%), 4052 (GO: 13.48%), and 16,057 (Interpro: 53.42%) unigenes were annotated (Table 1**)**. With functional annotation results, 24,097 CDS were detected (Additional file 4: Table S4). Based on NR annotation, the distribution of annotated species was 60.79% *B. dothidea* unigenes (14,603 unigenes) with a high match to *Macrophomina phaseolina* MS6 species genome [37], 20.09% (4826 Unigenes) with *Neofusicocum parvum* UCRNP2, and 1.63% (391) with *Coniosporium apollinis* CBS 100,218 (Table 2).

**Table 1:** Summary statistics of the functional annotation of all unigenes from *B.dothodea* strains in public data bases

Public database	Number of unigenes	Percentage (%)
Nr-Annotated	24,024	79.93%
Nt-Annotated	13,501	44.92%
Swissprot-Annotated	18,859	62.74%
KEGG-Annotated	19,722	65.61%
COG-Annotated	15,159	50.43%
Interpro-Annotated	16,057	53.42%
GO-Annotated	4052	13.48%
Annotated in at least one database	24,836	82.63%
All Unigenes	30,058	100%

**Table 2:** *B.dothidea* unigenes distribution in the top10 species

Species	Gene numbers	Percentage
*Macrophomina phaseoa* MS6	14,603	60.78%
*Neofusicoccum parvum* UCRNP2	4826	20.09%
*Coniosporium apolis* CBS 100218	391	1.63%
*Beauveria bassiana* D1–5	182	0.76%
*Endocarpon pusillum* Z07020	101	0.42%
*Pestalotiopsis fici* W106–1	98	0.41%
*Glarea lozoyensis* ATCC 20868	95	0.40%
*Nectria haematococca mp*VI 77–13-4	70	0.29%
*Phaeosphaeria nodorum* SN15	67	0.28%
*Setosphaeria turcica* Et28A	59	0.25%

**Fig. 3:**
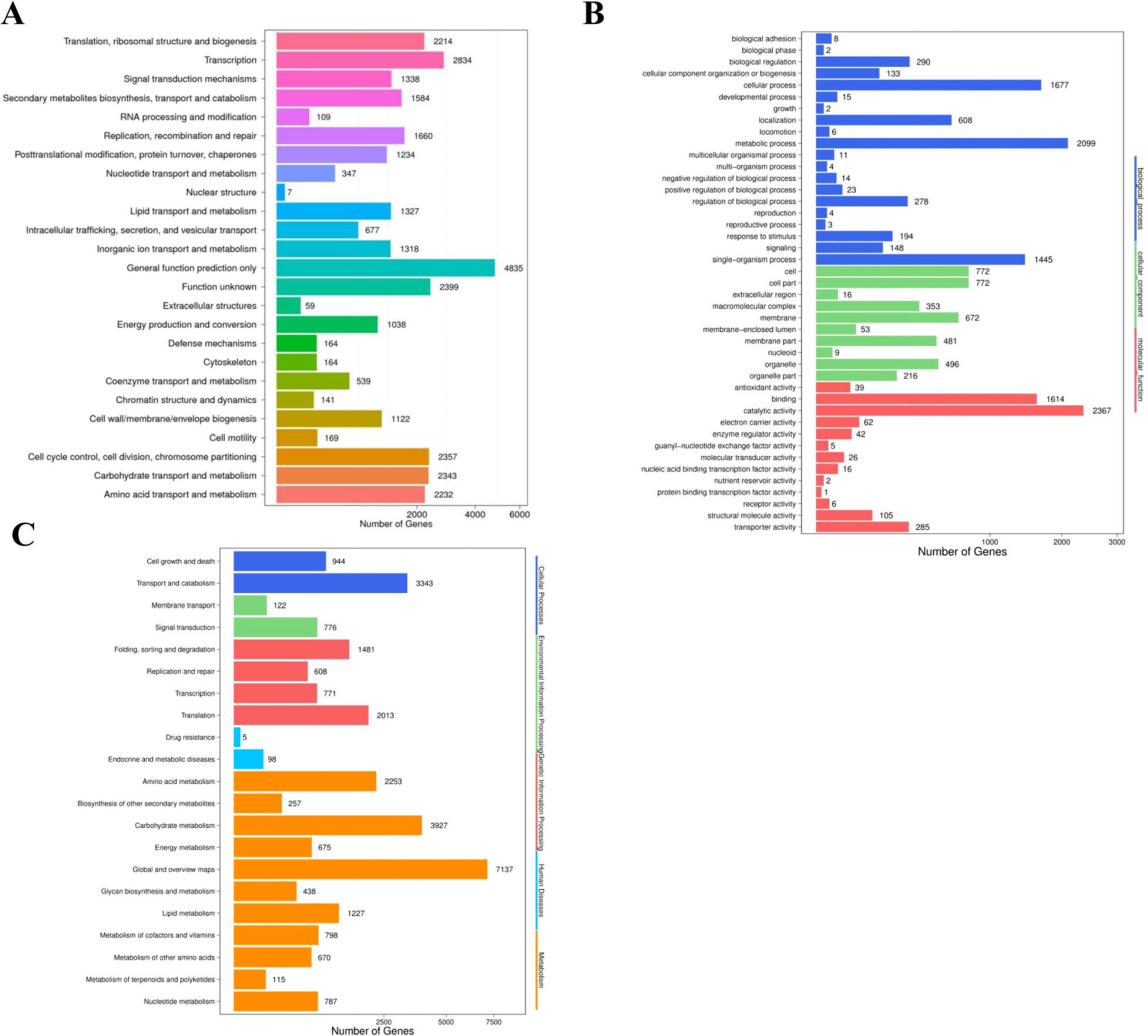
Functional distribution of COG annotation **a** GO annotation **b** and KEGG annotation **c** from *B.dothidea* unigenes. X axis represents the number of unigenes. Unigenes were grouped into 25 KOG categories in COG functional classification

The unigenes functional annotation in COG, GO, and KEGG were determined and classified, and are summarized in Fig. 3. In the COG classification, 15,159 unigenes (50.43%) were divided into 25 functional groups: ‘General function prediction only’ (4835 genes, 31.89%), representing the largest group, followed by ‘transcription’ (2834 genes, 18.7%), ‘function unknown’ (2399 genes, 15.8%), ‘replication, recombination, and repair’ (1660 genes, 10.95%), ‘signal transduction mechanism’ (1338 genes, 8.83%), and ‘defense mechanism’ (164 genes, 1.08%). These are summarized in Fig. 3a. The GO classification separated the 4052 unigenes (13.48%) into 43 functional groups representing biological processes, cellular component, and molecular function ontologies. In the biological processes group, ‘metabolic processes’ (2099 genes) and ‘cellular processes’ (1677 genes) were the top two GO terms. In the molecular function group, the top three GO terms were related to ‘catalytic activity’ (2367 genes), ‘binding’ (1614 genes), and ‘transporters activity’ (285 genes). A detailed analysis of the cellular component group showed ‘cell part’ (772 genes), ‘cell’ (772 genes), ‘membrane’ (672 genes), ‘organelle’ (496 genes), ‘membrane part’ (481 genes), and ‘macromolecular region part’ (353 genes) were highly represented (Fig. 3b). Searching the KEGG database revealed that 19,722 unigenes (65.61%) matched to KEGG pathways. The assembled unigenes were assigned to five specific functional categories, including cellular processes, environmental information processing, genetic information processing, metabolism, and organism systems. The most represented KEGG function category was the “global an overview maps” (7138 unigenes) and the ‘carbohydrate metabolism’ (3927 unigenes), belonging to the metabolism cluster. ‘Signal transduction’ (776 unigenes) and ‘membrane transport’ (122 unigenes) were annotated. ‘Drug resistance’ (5 unigenes) was the smallest group (Fig. 3c). These results reveal that *B. dothidea* infection with mycovirus mainly involve the metabolism pathway.

### Differential expression profiles of mRNAs from *B. dothidea* strains infection with mycovirus

**Fig. 4:**
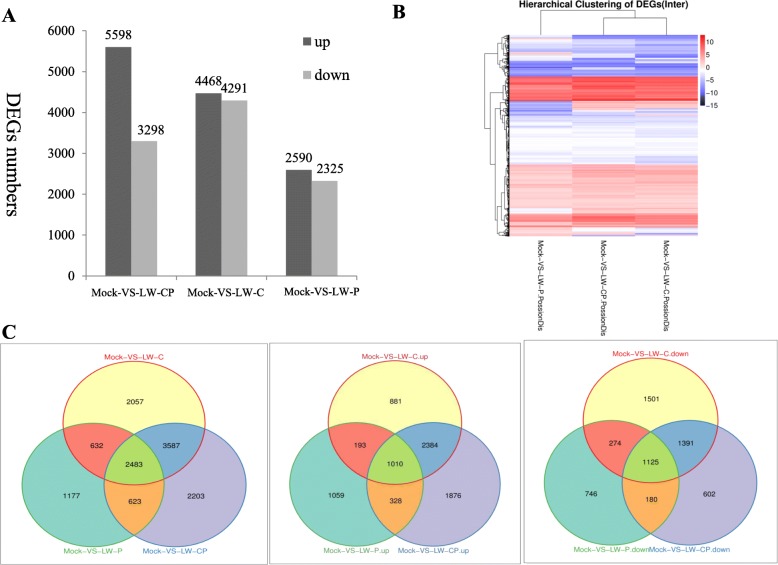
*B. dothidea* strains differentially expressed genes and cluster analysis of DEGs in response to single or mixed mycovirus chrysovirus 1 (BdCV1) and parititvirus 1 (BdPV1). **a** Number of DEGs: Blank and grey indicate the number of up-regulated and down-regulated genes, respectively. **b** Heat map of hierarchical clustering of DEGs. X-axis represents each sample: LW-CP, LW-C, and LW-P. Y-axis represents DEGs. Coloring indicates fold change (high: red, low: blue). **c** Venn diagrams illustrate the number of all up-regulated and down-regulated differentially expressed genes

Transcriptional changes in fungal genes following mycovirus infection have been little reported [17, 18, 22]. Here, we examined genome-wide transcriptional differences in *B. dothidea* expression in response to mycovirus infection. The number of up-regulated genes ranged from 2590 to 5598 genes while the number of down-regulated genes ranged from 2325 to 4291, at a cut-off of FDR < 0.001 and |log 2 (fold change)| > 1 (Fig. 4a). The number of DEGs was the highest in LW-CP (In total of 8896 genes, 5598 up-regulated and 3298 down-regulated), LW-C (8,759 genes, 4468 up-regulated and 4291 down-regulated), and lowest in LW-P (4,915 genes, 2590 up-regulated and 2325 down-regulated), indicating that many *B. dothidea* genes are regulated by mycovirus infection. Representative *B. dothidea* genes that show significant differential gene expression with functional annotation are summarized in Table 3. In addition, we detected the expression of genes involved in gene silencing pathways. These were up-regulated in response to mycovirus infection, such as argonaut-like genes (Ago), dicer-like genes (Dicer), and RNA-directed RNA polymerase (RdRp) genes (Additional file 5: Table S5). Genes annotated heat shock protein (Hsp)-related proteins involved in ‘response to stimulus’ pathway were also up-regulated (Additional file 6: Table S6). The above results reveal that the numbers of up-regulated transcripts were more prevalent than down-regulated transcripts. Notably, more genes were differentially expressed under BdCV1 compared to BdPV1. A hierarchical clustering of the mycovirus-responsive transcriptome demonstrates the significant similarities and differences in gene expression profiles among the three groups (LW-CP, LW-C, and LW-P; Fig. 4b). We next compared DEGs to determine the numbers of mycovirus-specific or commonly expressed genes in the three groups (Fig. 4c). A total of 2483 genes were identified in the three groups, revealing a potentially critical biological process. The number of specifically expressed genes in each mycovirus-infected sample was 2203 (1876 up-regulated and 602 down-regulated) for LW-CP, 2057 (881 up-regulated and 1501 down-regulated) for LW-C, and 1177 (1059 up-regulated and 746 down-regulated) for LW-P. Although many DEGs were mycovirus-specific, 2483 DEGs were common to LW-CP, LW-C, and LW-P, suggesting that they could be involved in stress response. The *B. dothidea* transcriptome analysis revealed that many host genes responsive to viral infection were common and mycovirus-specific expression [17, 38]. Increasing the stringency of differentially expressed genes progressively up to FDR < 0.001 and log_2_FC ≥ 2 (FC ≥ 4) also reveals a high number of significantly expressed transcripts (Additional file 7: Figure S7). DEGs were the highest in LW-CP (5313 genes) and the lowest in LW-P (2,738 genes). Furthermore, the hierarchical clustering and Venn diagram analyses demonstrate a similar trend with a cut-off of FDR < 0.001 and log_2_FC > 1(FC > 2) in the gene expression profiles among the three groups.

**Table 3:** Annotation of differentially expressed genes from *B.dothidea* strains in NR database

Gene ID	Length (nt)	Expression				log2FC			Regulation model	Nr-Annotation
		Mock	LW-CP	LW-C	LW-P	LW-CP /Mock	LW-C/Mock	LW-P/Mock		
CL1433.Contig1_All	2180	1.18	8.26	5.97	4.99	2.81	2.34	2.08	Up	putative amino acid permease protein
CL1608.Contig2_All	3553	3.65	35.67	30.32	12.37	3.29	3.05	1.76	Up	Sugar/inositol transporter
CL1856.Contig1_All	6267	0.01	1.9	0.18	0.59	7.57	4.17	5.88	Up	Kelch repeat type 1
CL2868.Contig1_All	3689	0.01	2.98	6.95	0.46	8.22	9.44	5.52	Up	Major facilitator superfamily
Unigene8027_All	1901	6.98	52.68	30.36	25.3	2.92	2.12	1.86	Up	GTP-binding domain HSR1-related protein
CL1210.Contig8_All	2904	0.01	5.08	1.23	1.03	8.99	6.94	6.69	Up	putative c6 transcription factor protein
CL1218.Contig1_All	3928	0.01	6.8	1.13	0.65	9.41	6.82	6.02	Up	Acyltransferase ChoActase/COT/CPT
CL1266.Contig2_All	1880	2.64	9.75	12.76	5.98	1.88	2.27	1.18	Up	CMP/dCMP deaminase zinc-binding protein
CL1272.Contig4_All	6904	0.01	6.24	6.28	2.32	9.29	9.29	7.86	Up	AIG1-like protein
CL1316.Contig1_All	1801	3.17	12.97	6.53	23.62	2.03	1.04	2.9	Up	Glutathione S-transferase transferase
CL1837.Contig3_All	1879	0.29	5.12	2.26	1.49	4.14	2.96	2.36	Up	Hrf1
CL2854.Contig1_All	5786	0.63	3.61	2.03	2.25	2.52	1.69	1.84	Up	Dopey
CL4079.Contig1_All	5893	0.01	1.48	1.12	0.28	7.21	6.81	4.81	Up	Putative ABC transporter protein
CL4156.Contig1_All	3680	0.01	16.2	4.06	1.68	10.66	8.67	7.39	Up	putative ubiquitin carboxyl-terminal hydrolase protein
CL54.Contig2_All	3595	0.01	4.58	1.8	0.64	8.84	7.49	6	Up	Chitin synthase
CL558.Contig1_All	2880	0.5	2.84	1.8	1.55	2.51	1.85	1.63	Up	Amino acid transporter transmembrane
Unigene1080_All	1950	1.89	7.58	5.71	7.35	2	1.6	1.96	Up	Carboxylesterase type B
Unigene5678_All	2696	5.48	18.76	17.01	21.07	1.78	1.63	1.94	Up	hypothetical protein MPH_13029
CL1188.Contig4_All	4955	9.3	2.35	0.89	1.33	−1.98	−3.39	−2.81	Down	Mg^2+^ transporter protein CorA-like/Zinc transport protein, partial
CL1201.Contig1_All	1401	294.62	87	45.55	103.27	−1.76	−2.69	− 1.51	Down	Ctr copper transporter
CL1240.Contig1_All	1071	1.84	0.01	0.01	0.29	−7.52	−7.52	−2.67	Down	Hypothetical protein CFIO01_01894
CL4468.Contig1_All	4397	74.92	25.48	30.42	27.28	−1.56	−1.3	−1.46	Down	Anoctamin/TMEM 16
Unigene1795_All	1309	5.33	1.8	1.29	0.87	−1.57	−2.05	−2.62	Down	Glycoside hydrolase family 5
Unigene2947_All	1216	2.88	0.9	0.66	0.51	−1.68	−2.13	−2.5	Down	ATPase P-type K/Mg/Cd/Cu/Zn/Na/Ca/Na/H-transporter
Unigene3433_All	972	98.16	1.33	0.56	1.66	−6.21	−7.45	−5.89	Down	hypothetical protein MPH_09082
Unigene4109_All	3383	15.54	0.83	1.42	3.01	−4.23	−3.45	−2.37	Down	Integral membrane protein
Unigene5131_All	961	1090.02	8.49	3.77	3.46	−7	−8.18	−8.3	Down	hypothetical protein MPH_01777
Unigene64_All	3669	4.14	0.93	0.44	1.16	−2.15	−3.23	−1.84	Down	Fungal chitin synthase
Unigene924_All	2470	10.62	2.61	1.47	1.32	−2.02	−2.85	−3.01	Down	Ferric reductase transmembrane component 4 precursors

### Validation of mRNA transcriptome analysis data by RT-qPCR

To confirm RNA-Seq results, we selected nine genes and purified total RNAs from different biological samples. The results of RNA-Seq were highly consistent with those of RT-qPCR using specific primers (Additional file 8: Table S8). For example, the expression of CL1217.Contig1 and CL1346.Contig4_All were strongly down-regulated, and CL2349Contig4_All, CL51.Contig10_All, CL1218.Contig1_All, Unigene3107_All, CL4042.Contig3_All, Unigene4082_All, and CL5019.Contig2_All were up-regulated in response to mycoviruses, which were all confirmed by RT-qPCR. This reveals that the transcript patterns by RT-qPCR are consistent with the RNA-Seq data analysis; however, the extent of differential expression differed (Table 4).

**Table 4:** qPCR analysis of *de novo* sequencing differentially expressed genes from *B.dothidea* across the three groups of LW-CP, LW-C, and LW-P

Gene ID	LW-CP		LW-C		LW-P		Regulation	Function (Nr)
	log2FC	RT-qPCR	log2FC	RT-qPCR	log2FC	RT-qPCR		
CL2349.Contig4_All	4.32	3.5	3.51	1.21	1.64	3.14	Up	Aa Family ATPase
CL51.Contig10_All	4.86	1.2	4.00	0.01	4.25	1.87	Up	putative cytoskeleton organisation protein
CL1218.Contig1_All	9.41	1.08	6.82	0.78	6.02	1.69	Up	Acyltransferase Choactase/COT/CPT
Unigene3107_All	4.89	1.41	3.95	0.54	/	1.98	Up	Ribonuclease III
CL4042.Contig3_All	6.04	2.7	6.23	1.48	6.73	2.28	Up	HR1 repeat rho-binding protein
Unigene4082_All	2.53	3.38	1.94	1.78	/	1.08	Up	Hypothetical protein MPH_05438
CL5019.Contig2_All	8.19	4.48	8.68	2.10	6.91	1.97	Up	Hypothetical protein MPH_07087
CL1217.Contig1_All	−2.44	−1.67	−1.82	−0.15	−1.29	−0.13	Down	Putative ABC transporter
CL1346.Contig4_All	−2.13	−1.61	− 2.39	−2.19	− 1.31	−0.76	Down	Cytochrome P450

**Note: “**/**”** indicated no significantly differential expression

### Effect of mycovirus infection on expression of predicted transcription factors

Because many fungal genes are unknown, only a limited number of transcription factors (TFs) were annotated [17, 18, 22]. In this study, 1083 TFs belonging to 18 families, including Zn2Cys6 (684 TFs) and C2H2 zinc finger (165 TFs), were predicated from *de novo* transcriptome data, many of which were differentially expressed in response to chrysovirus 1 BdCV1 and partitivirus 1BdPV1 infection. In total, 11 putative TF families were differentially expressed in response to different mycovirus infections. One hundred twenty eight TFs were commonly identified in all three mycovirus-infected groups by Venn diagrams. Up-regulated TFs were more numerous than down-regulated TFs in the LW-CP and LW-P groups, while more numerous TFs were down-regulated in the LW-C group. We further examined the portion of TF families enriched in DEGs. The Zn2Cys6 family was the dominant TF family followed by C2H2 zinc finger, MYB, and bHLH (Table 5; Additional file 9: Table S9 and Additional file 10: Figure S10).

**Table 5:** Differentially expressed TFs from *B. dothidea* strains belonging to 11 representative TF families were predicted in the three groups of LW-CP, LW-C, and LW-P, respectively

TF family	LW-CP		LW-C		LW-P	
	up	down	up	down	up	down
LIM	5	4	2	2	1	2
FHA	11	1	7	1	3	3
Alfin-like	2	3	2	2	0	3
Zn-clus	119	132	98	166	92	82
BSD	1	0	1	0	0	0
C3H	5	3	3	4	2	1
C2C2-GATA	0	1	0	2	1	0
MYB	12	6	9	6	7	4
bZIP	0	0	0	1	1	0
bHLH	7	3	8	5	6	1
C2H2	43	32	28	45	23	30
Total	205	185	158	234	136	126

### GO enrichment analysis of mycovirus-responsive unigenes from *B. dothidea* strains

**Fig. 5:**
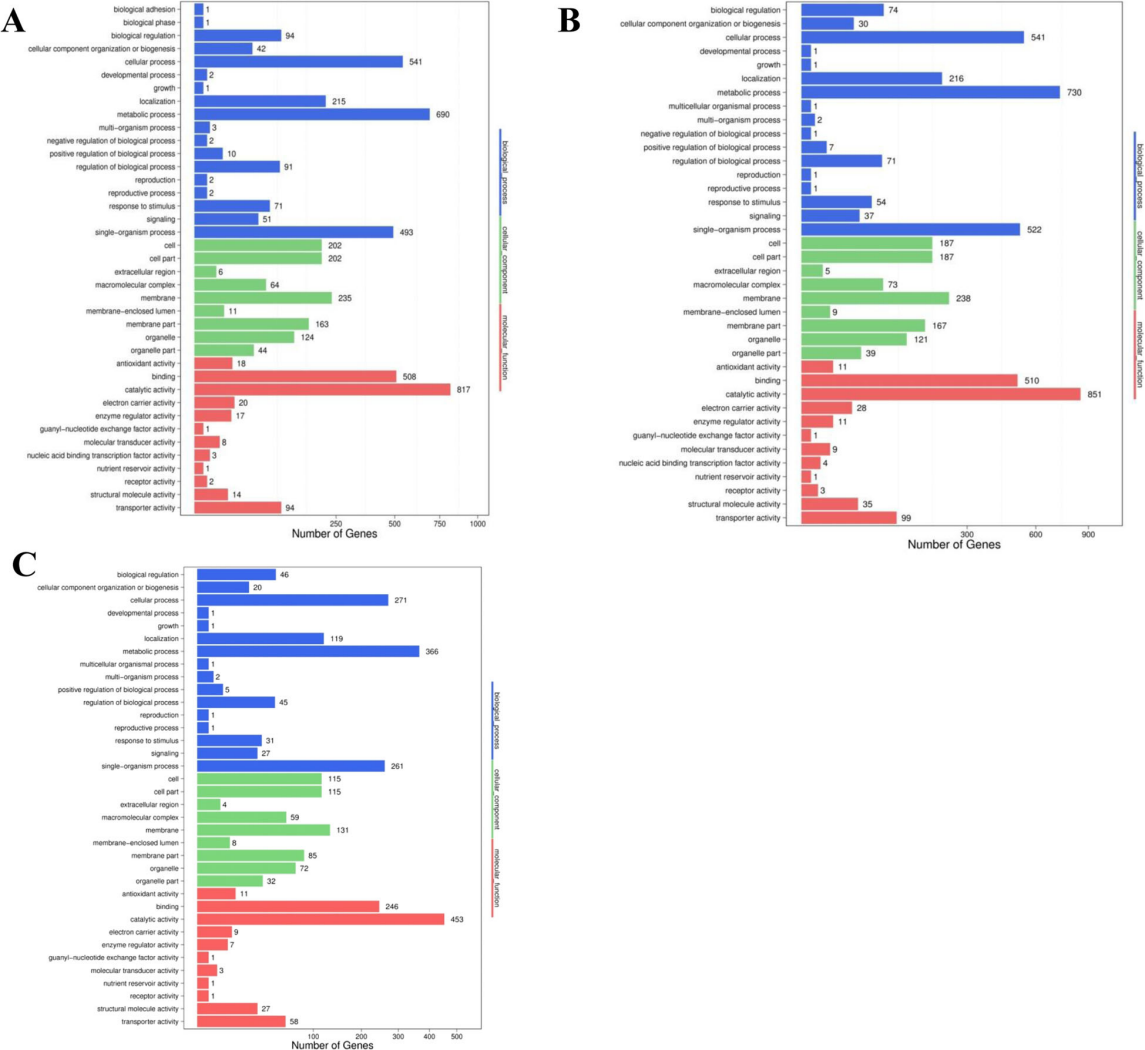
Gene Ontology (GO) classification analyses of DEGs across three groups: LW-CP **a** LW-C **b** and LW-P **c** X axis represents number of DEGs; Y axis represents GO term

To determine whether the mycovirus-responsive genes are involved in specific pathways, a functional classification of the DEGs was performed by GO enrichment analysis. By comparing LW-CP versus Mock, 4866 DEGs were functionally assigned to the relevant terms in three categories including biological processes, cellular component, and molecular function. The GO terms ‘metabolic process’ (GO: 0008152, 690 genes) in biological process, ‘catalytic activity’ (GO: 0003824, 817 genes) in molecular function, and ‘membrane’ (GO: 0016020, 235 genes) in the cellular component category were highly enriched (Fig. 5a).

In the LW-C group, 4879 DEGs were functionally assigned to: ‘metabolic process’ (GO: 0008152, 730 genes), followed by ‘cellular process’ (GO: 0009987, 541) in biological process. The ‘catalytic activity’ (GO: 0003824, 851 genes) in molecular function, were highly enriched. In the cellular component category, the ‘membrane’ (GO: 00016020, 238 genes), ‘integral component of membrane’ (GO: 00016021, 159 genes), and ‘intrinsic component of membrane’ (GO: 0031224, 238,159 genes) were significantly enriched (*p*-value is set as less than 0.05). In addition, other terms including ‘response to ‘stimulus’ annotated genes such as heat shock protein (Hsp)-related proteins, ‘Signaling’ and ‘transporter activity’ related to pathways involved in mycovirus-associated response were also enriched (Fig. 5b; Additional file 6: Table S6).

In the LW-P group, 2636 DEGs were functionally assigned. It is similar to the above two groups, such as ‘cell process’ (GO: 0009987, 271) in biological process, ‘catalytic activity’ (GO: 0003824, 453 genes) in molecular function, and ‘membrane’ (GO: 0016020, 131) in the cellular component category were highly enriched, respectively (Fig. 5c; Additional file 11: Figure S11).

### KEGG pathway analysis of mycovirus-responsive genes from *B. dothidea* strains

**Fig. 6:**
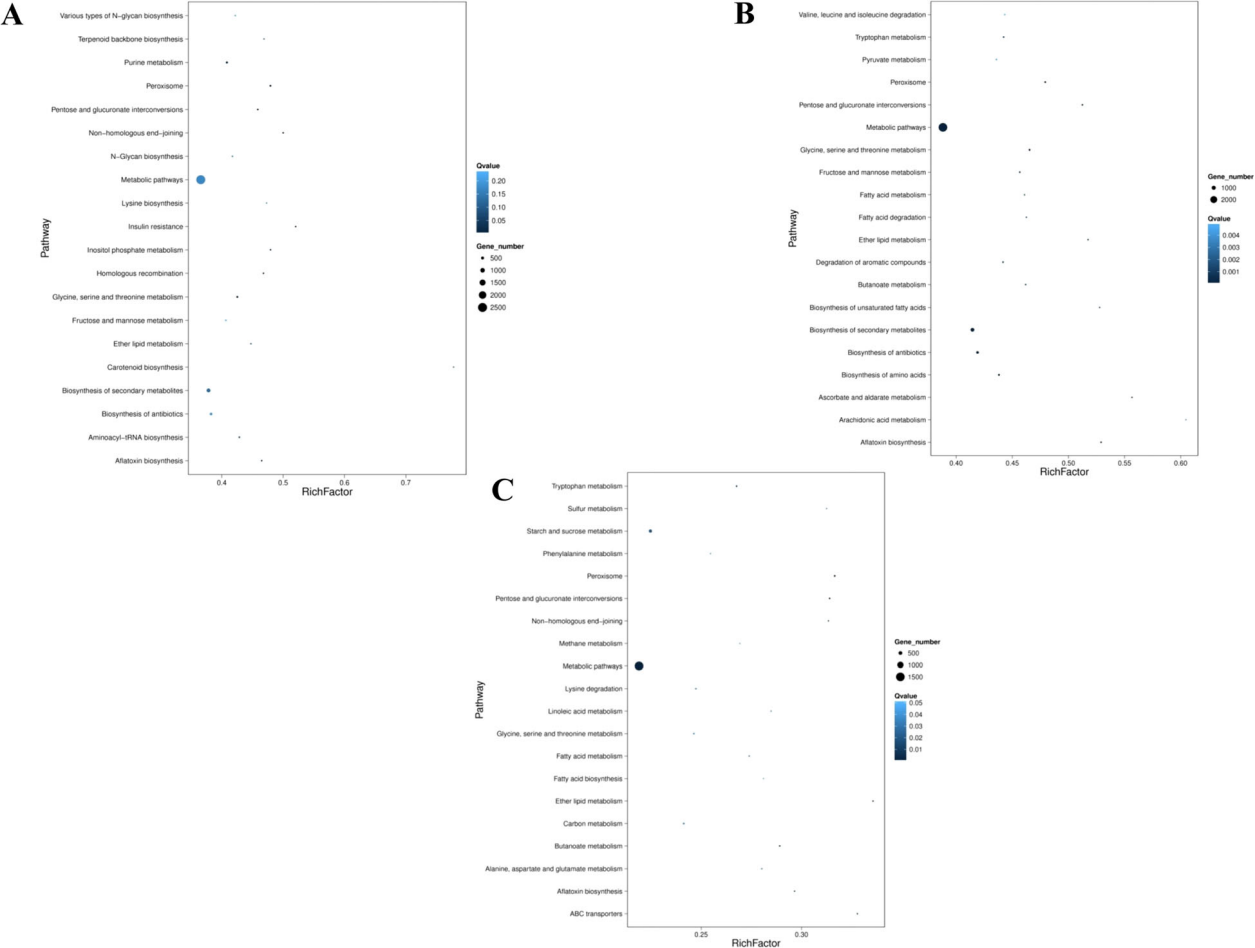
KEGG pathway classifications of functional enrichment for DEGs in *B. dothidea* strains infection with mycovirus in the three groups of LW-CP **a** LW-C **b** and LW-P **c** respectively. The 20 top enriched KEGG pathways based on DEGs are listed. X axis represents enrichment factor. Y axis represents KEGG pathway name. Color shows Q-value (high: light blue, low: dark blue), the lower Q-value represents a degree of enrichment that is more significant. The size of dot represents the number of DEG (large size of dot: more DEGs; small size: less DEGs)

To elucidate the biological processes, DEGs from *B.dothidea* strains regulated by mycovirus infection, and KEGG pathway were analyzed. The top 20 enriched pathways are summarized from the 3 groups. In the LW-CP group, 2554 DEGs were significantly enriched (corrected *p*-value < 0.05) in ‘metabolic pathways’ (PATH: ko01100), and 830 DEGs were related to ‘biosynthesis of secondary metabolites’ (Fig. 6a). However, this is approximately 36 and 12% of the total genes involved in ‘metabolic pathways’ and ‘biosynthesis of secondary metabolites’, respectively (Additional file 12: Table S12A). In the LW-C group, similar to the results of LW-CP, genes associated with ‘metabolic pathways’ (PATH: ko01100) and ‘biosynthesis of secondary metabolites’ (PATH: ko01110) (Fig. 6b), approximately 39 and 13% of the DEGs, respectively, were significantly enriched (Additional file 12: Table S12B). ‘Stach and sucrose metabolism’ (PATH: ko00500) related to carbohydrate metabolism, ‘Glycerophospholipid metabolism’ (PATH: ko00564) related to lipid metabolism, and ‘Indole diterpene alkaloid biosynthesis’ (PATH: ko00403) related to biosynthesis of other secondary metabolites, were all significantly enriched. Lastly, MAPK signaling pathway of signal transduction (PATH: ko04011) and ABC transporters of membrane transport (PATH: ko02010) were enriched. In the LW-P group, more DEGs were enriched in the ‘metabolic pathways’ and ‘Starch and sucrose metabolism’ (PATH: ko00500), with approximately 39 and 11% of the DEGs significantly enriched among the DEG, respectively (Fig. 6c). In addition, ‘peroxisome’ (PATH: ko04146), with functional classes related to transport and catabolism; ‘ABC transporters’ (PATH: ko02010), involved in membrane transport; and ‘ribosome’ (PATH: ko03010), involved in translation, were also enriched with approximately 2.7, 1, and 2.6% of the DEGs significantly enriched, respectively (Additional file 12: Table S12C).

From the above functional analysis, the DEGs associated with metabolic pathways, transport and catabolism, membrane transport, transcription, and signal transduction are presumed to play a role in the interaction of fungal defense and mycovirus counter defense strategies.

## Discussion

In this report, the transcriptome of *B. dothidea* strains was sequenced using the Illumina platform. A total of high-quality 18.87 Gb bases were obtained. We then assembled 4 fungi samples occurred in *B. dothidea* infection with mycovirus and obtained 30,058 Unigenes (≥200 bp). The N50 and GC content of unigenes was 3338 bp and 56.32%, respectively. We then annotated the unigenes using 7 functional databases. 24,836 (82.63%) unigenes were annotated with at least one functional database (Tables 1 and 2; Figs. 2 and 3). To the best of our knowledge, this is the first large-scale characterization of the *B. dothidea* genome at transcriptome level. Our results lay the foundation for further genomics research in *Botryosphaeria* species.

Previous research has demonstrated that mycovirus infections change the expression of a broad range of genes and cause hypovirulence or phenotypic alterations in the fungal host except plant [17–19, 22, 38, 39]. *C. parasitica* infected with Cryphonectria Hypovirus 1 (CHV1) is used as a model for studies on virus/host interaction. cDNA library analysis of differentially expressed genes involved viral replication, transmission, host growth, development, and defense. The pro1 and CpBir1 genes have important biological functions for hypovirus and chestnut blight fungus host [17, 40, 41]. In our study, RNA-Seq-based genome-wide expression profiling analysis in response to single or mixed mycovirus chrysovirus 1(BdCV1) and partitivirus 1 (BdPV1) infection was performed. The expression patterns of 9 putative genes involved in mycovirus stress measured by quantitative real-time PCR were consistent with their transcript changes as identified by RNA-seq (Table 4). It revealed specific and common alterations in host gene transcript accumulation following infection of *B. dothidea* by BdCV1 and BdPV1 [17]. As expected, more transcriptional changes occurred in response to the hypovirulent LW-CP and LW-C compared to the LW-P (Table 3; Fig. 4), revealing that chrysovirus 1(BdCV1) would have a stronger effect than partitivirus 1 (BdPV1) on the *B. dothidea* transcriptome, and gene expression changes in transcriptome caused by hypovirulent and non-hypovirulent mycoviruses were related to the observed host phenotypes and pathogenicity [17, 18]. Meanwhile, changes in differential accumulation of BdCV1 and BdPV1 in *B. dothidea* strains were demonstrated that the accumulation level of BdPV1 decreased by co-infection with BdCV1 (Fig. 1). It revealed that the BdCV1 and hypovirulent determinants may inhibit the expression level of BdPV1, prediction that it is the main reason to induce to the phenotype and hypovirulence of *B. dothidea* LW-1 strain (Figs. 1 and 4), which need to be further verified.

It is known that transcription factors play important roles in fungi response to mycovirus. Mycovirus can activate TFs expression differentially [18]. In this study, 1083 transcription factors classified into 18 families were predicted. The above data revealed many TFs from *B. dothidea* were expressed differentially among the mycoviruses, especially Zn2Cys6 and C2H2 zinc finger families (Table 5). This corroborates the results from the *F. graminearum* transcriptome, whose differentially expressed TFs included fungal-specific and dominant Zn2Cys6 and the C2H2 zinc finger members involved in transcriptional regulation [18, 22, 42, 43]. The results demonstrate that TFs are down-regulated in response to BdCV1 in LW-C, revealing that BdCV1 inhibits TFs expression in *B. dothdiea* (Table 5; Additional file 9: Table S9). In addition, a class of heat shock proteins (HSPs) was up-regulated in response to mycovirus infection (Additional file 6: Table S6), which supports the speculation that HSPs homologues are influenced by mycovirus [17].

To better survey the biological behavior of defense response, it is necessary to understand the functional distribution of these DEGs in *B. dothidea* following mycovirus infection based on the transcriptome and bioinformatics analysis [44–47]. Through the enriched GO terms analysis, it is also revealed that in biological processes, DEGs mainly distributed to metabolism. Differential expression of genes related to metabolism might be associated with the host phenotype [17, 39, 48]. In addition, GO terms for genes associated with signal transduction, including phosphatidylinositol signaling system and MAPK signaling pathway were more enriched in response to infection by BdCV1 compared to BdPV1. This reveals that it is necessary to determine the role of the MAPK signaling pathway in the regulation of mycovirus and host interactions [25]. Genes involved in membrane, oxidoreductase activity, RNA biosynthetic processing, and ribosomal assembly were enriched in *B. dothidea* following mycovirus infection (Fig. 5; Additional file 11: Table S11), which indicates the diversity of genes affected by mycoviral infection. Furthermore, KEGG pathway analysis uncovered DEGs involved in important pathways. A mass of metabolism pathways, both primary and biosynthesis of secondary metabolism, are identified and significantly enriched in response to mycovirus infection (Fig. 6; Additional file 12: Table S12). Similar metabolic pattern is exhibited in different fungi-virus combinations, which indicated these pathways play important role in fungi host in response to mycoviral infection [17, 18, 22]. In addition, ‘carbohydrate metabolism’, ‘lipid metabolism’, ‘membrane transport system’, ‘transport and catabolism’, ‘translation’ and ‘signal transduction’ which were highly enriched (Fig.6; Additional file 12: Table S12). It also provides insight into the various biological pathways associated with mycoviral infection with plant pathogenic fungi.

RNAi machinery is involved in the regulation of fungi and mycovirus infection by controlling endogenous and exogenous RNA [49]. Indeed, the biological functions of the RNA silencing pathway have been characterized in the *Neurospora crassa*, *Cryphonectria parasitaica*, *Rosellinia necatrix,* and *F. graminearum* [50–56]. As reported, FgAGO-1 and FgDICER-2 are responsible for hairpin RNA-triggered RNA silencing and related small interfering RNA accumulation in *F. graminearum* [18, 49, 51]. In this study, RNA interference components, including dicer-like (*Dicer*), argonaute-like (*Ago*), and *RdRp* genes in *B. dothidea* were expressed differentially in response to mycovirus infection (Additional file 5: Table S5). Small RNA sequencing demonstrates that microRNA exists and is expressed differentially in *B. dothidea* infection with mycovirus (not published). This suggests that *B. dothidea* possesses RNA silencing pathways for antiviral defense and endogenous gene regulation. These data indicate that mycovirus may activate host antiviral defense. Furthermore, it is necessary to determine and analyze the RNA silencing component responsible for transcriptional regulation and antiviral defense mechanism in *B. dothidea*.

## Conclusions

In this study, 30,058 unigenes were obtained from hypovirulent and non-hypovirulent *B. dothidea* strains infected with mycovirus by *de novo* assembly. To identify potential mycovirus-responsive genes, DEGs were screened and further validated by RT-qPCR. Hierarchical clustering, which identifies gene sets of significantly differentially expressed genes occurred in *B. dothidea* infection with mycovirus, was performed. The expression analysis demonstrates that hypovirulent mycoviruse chrysovirus 1 (BdCV1) have a stronger effect than non-hypovirulent mycoviruse patittivirus 1 (BdPV1) on the *B. dothidea* transcriptome. This data indicates that the phenotypes and pathogenicity observed for mycovirus-infected *B. dothidea* are correlated with the numbers of DEGs and mycovirus variety [17, 18]. In addition, we found that most *B. dothidea* TFs were differentially expressed by each mycovirus, suggesting that fungal TFs have important roles in the response to mycovirus infection. Gene ontology (GO) enrichment and KEGG functional pathway analysis revealed that differential expression mRNAs played important roles in regulating the complex biological processes involved in *B. dothidea* infection with mycovirus. The obtained transcriptome data can provide molecular genomics resource for further functional characterization analysis of *B. dothidea* in response to mycovirus infection.

## Additional files

Additional file 1: **Table S1.** *B.dothidea* transcriptome sequencing summary after filtering. (DOCX 258 kb) (DOCX 17 kb)

Additional file 2: **Table S2.** Quality metrics of transcripts and Unigenes from *B.dothidea* transcriptome sequencing. (DOCX 715 kb) (DOCX 18 kb)

Additional file 3: **Table S3.** The consistency of unigene sequences from PCR detection and cloning with *De novo* sequencing. (XLSX 46 kb) (DOCX 17 kb)

Additional file 4: **Table S4.** Quality metrics of predicated CDS from *B.dothidea* transcripts. (DOCX 17 kb)

Additional file 5: **Table S5.** Ago, Dicer and RdRp genes from *B.dothdiea* involved in post transcriptional gene silencing were expressed in response to mycovirus by *De novo* sequencing. (DOCX 18 kb) (DOCX 19 kb)

Additional file 6: **Table S6.** The genes annotated Heat shock protein (Hsp)-related proteins in database, were expressed differentially among LW-CP/Mock, LW-C/Mock and LW-P/Mock libraries, respectively. (DOCX 17 kb) (XLSX 24 kb)

Additional file 7: **Figure S7.** *B. dothidea* genes differentially expressed and cluster analysis of DEGs at FDR < 0.001 and log2FC ≥ 2 (FC ≥ 4) among LW-CP/Mock, LW-C/Mock and LW-P/Mock libraries, respectively. (A)Heatmap of hierarchical clustering of DEGs. X axis represents each comparing samples of LW-CP/Mock, LW-C/Mock and LW-P/Mock, respectively. Y axis represents DEGs. Coloring indicates fold change (high: red, low: blue). (B) The numbers of DEGs, Blank and gray indicate the numbers of up-regulated and down-regulated expression genes, respectively. (C) Venn diagrams illustrate the numbers of all, up-regulated and down-regulated differential expression genes, respectively. (DOCX 17 kb) (DOCX 569 kb)

Additional file 8: **Table S8.** The primers used for RT-qPCR expression analysis mRNAs from *B.dothidea*, BdCV1 and BdPV1 genes, respectively. (DOCX 18 kb)

Additional file 9: **Table S9.** The expressed change ratio (log2FC relative to the virus-free strain) of *B.dothidea* TFs in each mycovirus-infected sample. (XLSX 208 kb)

Additional file 10: **Table S10.** Transcription factor family classification for All-unigenes from *B.dothidea* strains (A), and Venn diagrams illustrating the numbers of TFs that were differentially expressed in subsets of the three virus-infected strains; (B).Total, up, and down indicate the total numbers of DEGs, the numbers of up-regulated DEGs, and the numbers of down-regulated DEGs, respectively. (DOCX 258 kb)

Additional file 11: **Figure S11.** Gene Ontology (GO) enrichment terms analysis of DEGs in responsive to LW-P showing the main enriched processes related to (A) Biological process, (B) Cellular component, (C) Molecular function. (DOCX 715 kb)

Additional file 12: **Table S12.** The results of KEGG pathway classification for DEGs from (A) LW-CP/Mock, (B) LW-C/Mock, and (C) LW-P/Mock libraries, respectively. (XLSX 46 kb)
