# Supplementary material for: De novo transcriptomic assembly and mRNA expression patterns of Botryosphaeria dothidea infection with mycoviruses chrysovirus 1 (BdCV1) and partitivirus 1 (BdPV1)
Source: Virol J. 2018 Aug 13;15:126. doi: 10.1186/s12985-018-1033-4 (PMC6088430; doi:10.1186/s12985-018-1033-4)
Supplement: Supplementary file 1 — Table S1. B.dothidea transcriptome sequencing summary after filtering. (DOCX 258 kb) (DOCX 17 kb) [file 12985_2018_1033_MOESM1_ESM.docx]

**Additional file 1: Table S1** *B.dothidea* transcriptome sequencing summary after filtering.

| **Strains** | **Total Raw Reads(Mb)** | | **Total Clean Reads(Mb)** | **Total Clean Bases(Gb)** | **Clean Reads Q20(%)** | **Clean Reads Q30(%)** | **Clean Reads Ratio (%)** |
| --- | --- | --- | --- | --- | --- | --- | --- |
| LW-C | | 48.44 | 47.65 | 4.77 | 98.64 | 96.38 | 98.37 |
| LW-CP | | 47.48 | 46.71 | 4.67 | 98.73 | 96.49 | 98.36 |
| LW-P | | 48.38 | 47.53 | 4.75 | 98.67 | 96.48 | 98.23 |
| Mock | | 47.72 | 46.84 | 4.68 | 98.65 | 96.50 | 98.15 |
|  |  | |  |  |  |  |  |

Q20: the rate of bases which quality is greater than 20.
