# Supplementary material for: De novo transcriptomic assembly and mRNA expression patterns of Botryosphaeria dothidea infection with mycoviruses chrysovirus 1 (BdCV1) and partitivirus 1 (BdPV1)
Source: Virol J. 2018 Aug 13;15:126. doi: 10.1186/s12985-018-1033-4 (PMC6088430; doi:10.1186/s12985-018-1033-4)
Supplement: Supplementary file 2 — Table S2. Quality metrics of transcripts and Unigenes from B.dothidea transcriptome sequencing. (DOCX 715 kb) (DOCX 18 kb) [file 12985_2018_1033_MOESM2_ESM.docx]

**Additional file 2: Table S2** Quality metrics of transcripts and Unigenes from *B.dothidea* strains transcriptome sequencing

| **Genes** | **Sample** | **Total Number** | **Total Length** | **Mean Length** | **N50** | **N70** | **N90** | **GC (%)** |
| --- | --- | --- | --- | --- | --- | --- | --- | --- |
| transcripts | LW-C | 26,461 | 37,805,678 | 1,428 | 2,523 | 1,744 | 696 | 56.42 |
|  | LW-CP | 30,707 | 52,558,770 | 1,711 | 2,891 | 2,003 | 943 | 56.28 |
|  | LW-P | 25,808 | 39,820,688 | 1,542 | 2,774 | 1,887 | 770 | 56.43 |
|  | Mock | 25,298 | 38,385,066 | 1,517 | 2,673 | 1,821 | 763 | 56.46 |
| Unigenes | LW-C | 23,056 | 35,441,886 | 1,537 | 2,546 | 1,771 | 775 | 56.46 |
|  | LW-CP | 27,554 | 50,599,534 | 1,836 | 2,927 | 2,039 | 1,015 | 56.3 |
|  | LW-P | 22,403 | 37,209,545 | 1,660 | 2,800 | 1,910 | 8,46 | 56.47 |
|  | Mock | 22,068 | 35,900,672 | 1,626 | 2,690 | 1,850 | 8,39 | 56.5 |
|  | All-Unigene | 30,058 | 63,980,206 | 2,128 | 3,338 | 2,328 | 1,225 | 56.32 |

N50: a weighted median statistic that 50% of the Total Length is contained in Unigenes or transcrips great than or equal to this value. GC (%): the percentage of G and C bases in all Unigenes or transcripts.
