# Supplementary material for: De novo transcriptomic assembly and mRNA expression patterns of Botryosphaeria dothidea infection with mycoviruses chrysovirus 1 (BdCV1) and partitivirus 1 (BdPV1)
Source: Virol J. 2018 Aug 13;15:126. doi: 10.1186/s12985-018-1033-4 (PMC6088430; doi:10.1186/s12985-018-1033-4)
Supplement: Supplementary file 3 — Table S3. The consistency of unigene sequences from PCR detection and cloning with De novo sequencing. (XLSX 46 kb) (DOCX 17 kb) [file 12985_2018_1033_MOESM3_ESM.docx]

**Additional file 3:** **Table S3** The consistency of unigene sequences from PCR detection and cloning with *De novo* sequencing

| Gene ID | Length (bp) | Forward primer (5'-3') | Reverse primer (5'-3') | PCR ID | PCR product size (bp) | Identity |
| --- | --- | --- | --- | --- | --- | --- |
| Unigene2632_All | 874 | CTCGTAGAGGCCCAAATCAGC | CTTAGGAAAACGCTTAACGCAGT | A1 | 696 | 98% |
| CL954.Contig5_All | 1023 | TACTACTTCGTCTCTCCCTAGGCA | TCGCATAGCCACTACTGCTC | A2 | 769 | 99% |
| CL1007.Contig1_All | 1274 | TCCATCCTCTCTCCCTCGAAC | CCCATCACATCCGCAATCTCC | A3 | 1,001 | 99.20% |
| CL2579.Contig2_All | 1452 | CCCAGAAAGCCCGAGCTAC | AACACGGACCAACCACTG | A4 | 1,013 | 99.90% |
| CL4812.Contig2_All | 1432 | CTGCACCAATTATGGCGGACT | TGACTCTCCGCAGTTGAACCC | A5 | 1,141 | 99.60% |
| Unigene 5720 | 1875 | AACGCAAGACTCTGGAAC | GAGCTTGGACGAGTACAT | A6 | 1,875 | 100% |
| CL1018.Contig5_All | 976 | TGACCTACATGGCAATGCT | GTTTCCCATTTCCACGTCT | A7 | 731 | 98.90% |
