# Supplementary material for: De novo transcriptomic assembly and mRNA expression patterns of Botryosphaeria dothidea infection with mycoviruses chrysovirus 1 (BdCV1) and partitivirus 1 (BdPV1)
Source: Virol J. 2018 Aug 13;15:126. doi: 10.1186/s12985-018-1033-4 (PMC6088430; doi:10.1186/s12985-018-1033-4)
Supplement: Supplementary file 4 — Table S4. Quality metrics of predicated CDS from B.dothidea transcripts. (DOCX 17 kb) [file 12985_2018_1033_MOESM4_ESM.docx]

**Additional file 4:** **Table S4** Quality metrics of predicated CDS from *B.dothidea* transcripts.

| Software | Total Number | | Total Length | | Mean Length | | N50 | N70 | N90 | | GC (%) |
| --- | --- | --- | --- | --- | --- | --- | --- | --- | --- | --- | --- |
| Blast | | 24,097 | | 30,421,020 | | 1,262 | 1,686 | 1,224 | | 693 | 57.51 |
| ESTScan | | 867 | | 297,300 | | 342 | 357 | 267 | | 213 | 55.79 |
| Overall | | 24964 | | 30,718,320 | | 1,230 | 1,674 | 1,212 | | 669 | 57.5 |
