# Supplementary material for: De novo transcriptomic assembly and mRNA expression patterns of Botryosphaeria dothidea infection with mycoviruses chrysovirus 1 (BdCV1) and partitivirus 1 (BdPV1)
Source: Virol J. 2018 Aug 13;15:126. doi: 10.1186/s12985-018-1033-4 (PMC6088430; doi:10.1186/s12985-018-1033-4)
Supplement: Supplementary file 5 — Table S5. Ago, Dicer and RdRp genes from B.dothdiea involved in post transcriptional gene silencing were expressed in response to mycovirus by De novo sequencing. (DOCX 18 kb) (DOCX 19 kb) [file 12985_2018_1033_MOESM5_ESM.docx]

**Additional file 5: Table S5** Ago, Dicer and RdRp genes from *B.dothdiea* involved in post transcriptional gene silencing were expressed in response to mycovirus by *De novo* sequencing

| Gene name | |  | LW-CP/Mock | | | LW-C/Mock | | | LW-P/Mock | | |
| --- | --- | --- | --- | --- | --- | --- | --- | --- | --- | --- | --- |
|  |  | Length  bp | Log2FC(LW-1/Mock) | p-value | Mode | Log2FC(LW-C/Mock) | p-value | Mode | Log2FC(LW-P/Mock) | p-value | Mode |
| Ago | Unigene2065_All | 4,505 | -0.219 | 1.43e-09 | / | -0.004 | 0.89 | / | 0.483 | 1.79e-51 | / |
|  | Unigene3249_All | 3,971 | -0.129 | 0.018 | / | -0.450 | 9.19e-155 | / | 0.580 | 8.04e-33 | / |
| Dicer | Unigene2349_All | 651 | 8.06 | 3.38e-06 | Up | / | / | / | / | / | / |
|  | Unigene3107_All | 860 | 4.89 | 1.27e-08 | Up | 3.95 | 0.0001 | Up | / | / | / |
|  | Unigene4240_All | 1,861 | 2.79 | 2.96e-11 | Up | 1.60 | 0.0018 | / | 0.097 | 0.85 | / |
|  | CL2827.Contig1_All | 1,064 | 2.98 | 6.37e-18 | Up | / | / | / | -6.71 | 0.0002 | Down |
|  | Unigene3648_All | 4,957 | 0.094 | 0.284 | / | -0.090 | 0.32 | / | 0.016 | 0.86 | / |
| RdRp | CL4691.Contig2_All | 4,700 | 0.26 | 0.0041 | / | 0.12 | 0.17 | / | 0.20 | 0.032 | / |
|  | CL2635.Contig3_All | 7,256 | 0.15 | 0.003 | / | -0.10 | 0.044 | / | -0.10 | 0.44 | / |
|  | CL2635.Contig4_All | 9,924 | 0.177 | 0.0005 | / | -0.015 | 0.75 | / | -0.015 | 0.75 | / |
