# Supplementary material for: De novo transcriptomic assembly and mRNA expression patterns of Botryosphaeria dothidea infection with mycoviruses chrysovirus 1 (BdCV1) and partitivirus 1 (BdPV1)
Source: Virol J. 2018 Aug 13;15:126. doi: 10.1186/s12985-018-1033-4 (PMC6088430; doi:10.1186/s12985-018-1033-4)
Supplement: Supplementary file 7 — Figure S7. B. dothidea genes differentially expressed and cluster analysis of DEGs at FDR < 0.001 and log2FC ≥ 2 (FC ≥ 4) among LW-CP/Mock, LW-C/Mock and LW-P/Mock libraries, respectively. (A)Heatmap of hierarchical clustering of DEGs. X axis represents each comparing samples of LW-CP/Mock, LW-C/Mock and LW-P/Mock, respectively. Y axis represents DEGs. Coloring indicates fold change (high: red, low: blue). (B) The numbers of DEGs, Blank and gray indicate the numbers of up-regulated and down-regulated expression genes, respectively. (C) Venn diagrams illustrate the numbers of all, up-regulated and down-regulated differential expression genes, respectively. (DOCX 17 kb) (DOCX 569 kb) [file 12985_2018_1033_MOESM7_ESM.docx]

**Additional file 7: Figure S7** *B. dothidea* genes differentially expressed and cluster analysis of DEGs at FDR<0.001 and log2FC≥2 (FC≥4) among LW-CP/Mock, LW-C/Mock and LW-P/Mock libraries, respectively.

(A)Heatmap of hierarchical clustering of DEGs. X axis represents each comparing samples of LW-CP/Mock, LW-C/Mock and LW-P/Mock, respectively. Y axis represents DEGs. Coloring indicates fold change (high: red, low: blue). (B)The numbers of DEGs, Blank and grey indicate the numbers of up-regulated and down-regulated expression genes, respectively. (C)Venn diagrams illustrate the numbers of all, up-regulated and down-regulated differential expression genes, respectively.


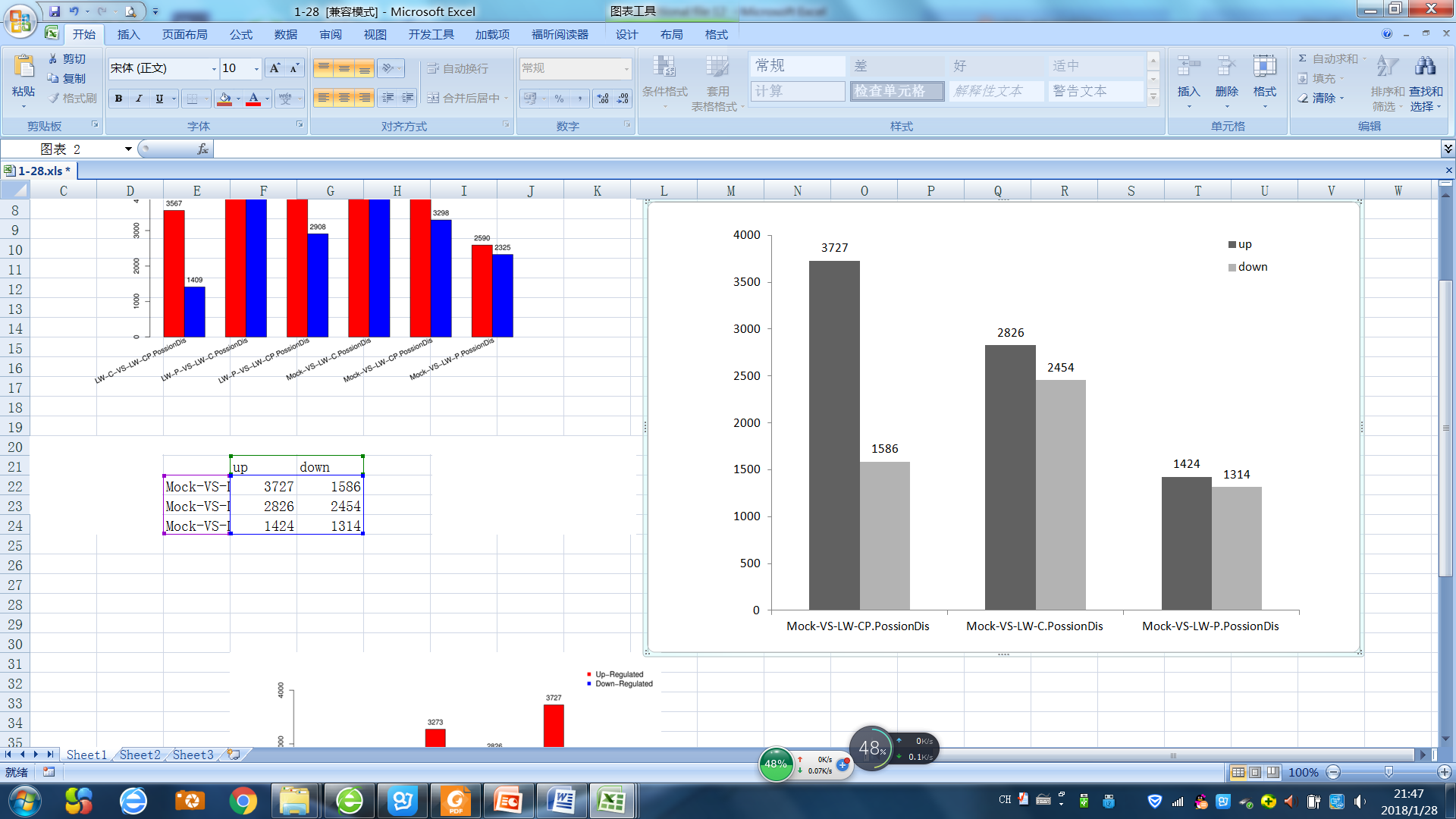

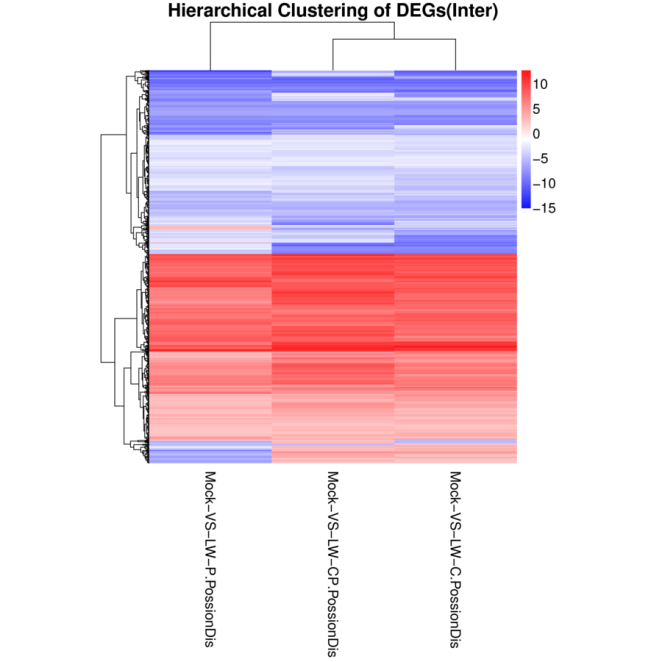

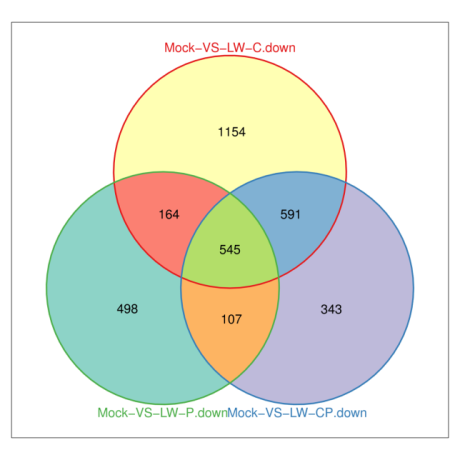

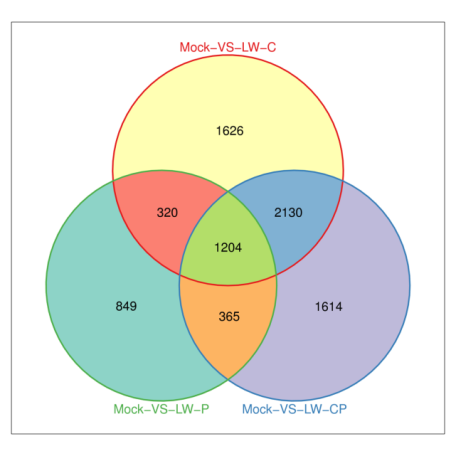

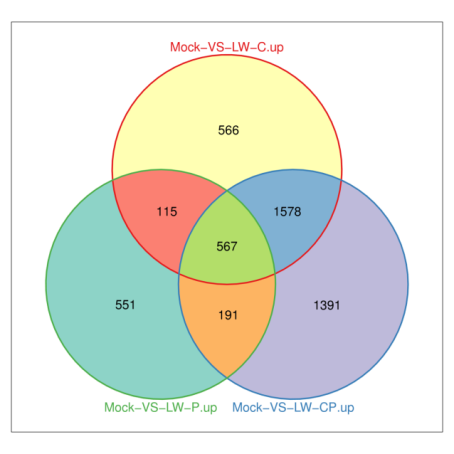


**B**

**C**

**A**
