# Supplementary material for: De novo transcriptomic assembly and mRNA expression patterns of Botryosphaeria dothidea infection with mycoviruses chrysovirus 1 (BdCV1) and partitivirus 1 (BdPV1)
Source: Virol J. 2018 Aug 13;15:126. doi: 10.1186/s12985-018-1033-4 (PMC6088430; doi:10.1186/s12985-018-1033-4)
Supplement: Supplementary file 8 — Table S8. The primers used for RT-qPCR expression analysis mRNAs from B.dothidea, BdCV1 and BdPV1 genes, respectively. (DOCX 18 kb) [file 12985_2018_1033_MOESM8_ESM.docx]

**Additional file 8: Table S8** The primers used for RT-qPCR expression analysis mRNAs from *B.dothidea*, BdCV1 and BdPV1 genes, respectively.

| Gene Name | Primer sequence(5′-3′) | PCR produces of Size of primers (bp) | |
| --- | --- | --- | --- |
|  | *B.dothidea* genes (mRNAs) | | |
| CL2349.Contig4_All | F-ATGCTTCGTTATACTACT | | 100 |
|  | R-GTGGCTATTGTTATATCG | |  |
| CL51.Contig10_All | F-GCAATTATCGGTTACCAT  R-GAATGAATCTCGTTGGAA | | 103 |
| CL1218.Contig1_All | F-GAAGAGACCATTGTTACA  R-GCGAATAGAGAAGACTAC | | 175 |
| Unigene3107_All | F-GTGCTCAGATATGTGTTG  R-TCGTCAAGAGTAGAGATG | | 120 |
| CL4042.Contig3_All | F-TAACTTAATTGCCGTCTGT  R-TGTACGATATTGATGAGGAAT | | 101 |
| Unigene4082_All | F-TTGTGGATGTTCTTAGACT  R-TTTCTCTTGGGACCATAG | | 111 |
| CL5019.Contig2_All | F-TGTTCATGTTAGCAGTAG  R-GTAAGGTTGTAAGGAGATA | | 151 |
| CL1217.Contig1_All | F-CGATAAGCATTTCAAGGT  R-CATAAAGGACAACATTCTATTC | | 102 |
| CL1346.Contig4_All | F-TGAGTGAAGTGGATATTG  R-TAGTCAGCAGATAGTAGA | | 101 |
| 18s | F-CTCAACACGGGGAAACTCAC  R-ATTTAGCAGGTTAAGGTCTCGTTC | | 144 |
|  | BdCV1 and BdPV1 | |  |
| BdCV1 *RdRp* | F-CGTCGTTCACCAACAGCATCC  R-ACTCGAACCCCATTACTTTACCG | | 186 |
| BdPV1 *RdRp* | F- GGTGATGAGGACATGTCGAG | | 144 |
|  | R- AAGGAAACCAACTGGACGAC | |  |
| BdCV1 *cp* | F-CATGAGCGCATCTGTCAACGTACC | | 131 |
|  | R-CATACAGCAACAACGCGGTTTCCA | |  |
| BdPV1 *cp* | F-GACACGACGTAGGACGAAGA | | 154 |
|  | R-ACGATCAATCCAAACGTTCA | |  |
