# Supplementary material for: De novo transcriptomic assembly and mRNA expression patterns of Botryosphaeria dothidea infection with mycoviruses chrysovirus 1 (BdCV1) and partitivirus 1 (BdPV1)
Source: Virol J. 2018 Aug 13;15:126. doi: 10.1186/s12985-018-1033-4 (PMC6088430; doi:10.1186/s12985-018-1033-4)
Supplement: Supplementary file 10 — Table S10. Transcription factor family classification for All-unigenes from B.dothidea strains (A), and Venn diagrams illustrating the numbers of TFs that were differentially expressed in subsets of the three virus-infected strains; (B).Total, up, and down indicate the total numbers of DEGs, the numbers of up-regulated DEGs, and the numbers of down-regulated DEGs, respectively. (DOCX 258 kb) [file 12985_2018_1033_MOESM10_ESM.docx]

**Additional file 10: Figure S10**. Transcription factor family classification for All-unigenes from *B.dothidea* strains (A), and Venn diagrams illustrating the numbers of TFs that were differentially expressed in subsets of the three virus-infected strains; (B).Total, up, and down indicate the total numbers of DEGs, the numbers of up-regulated DEGs, and the numbers of down-regulated DEGs, respectively.


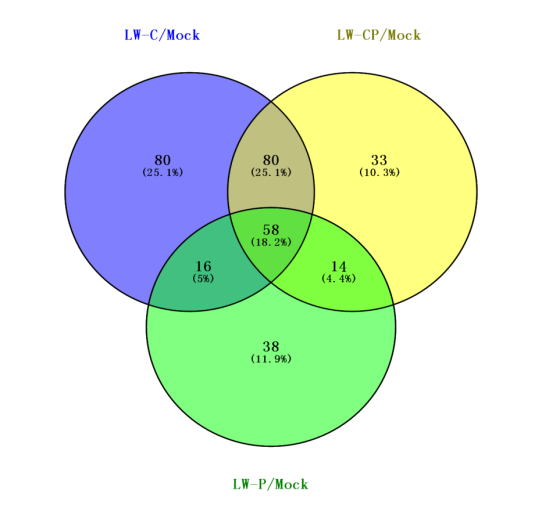

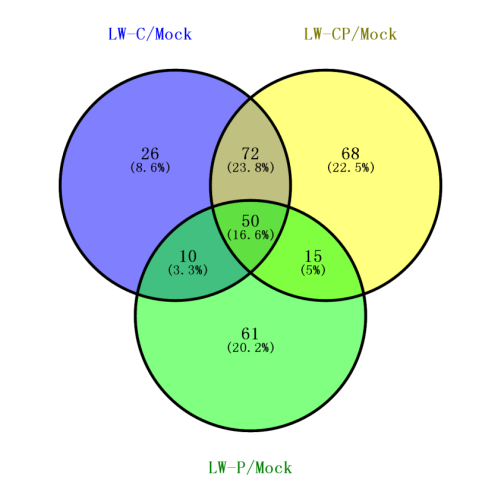

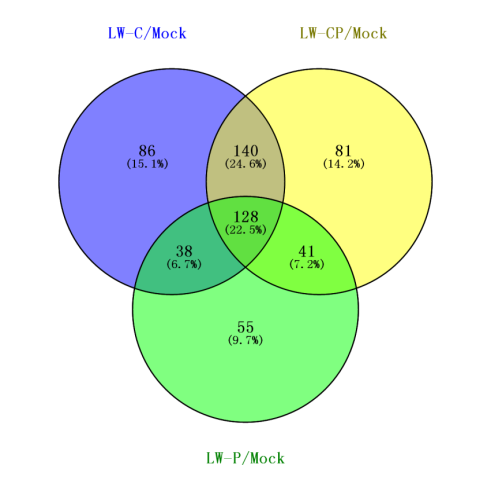

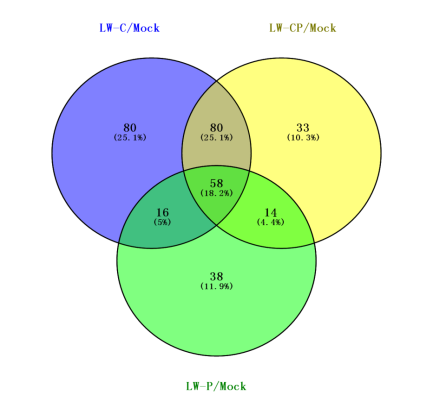

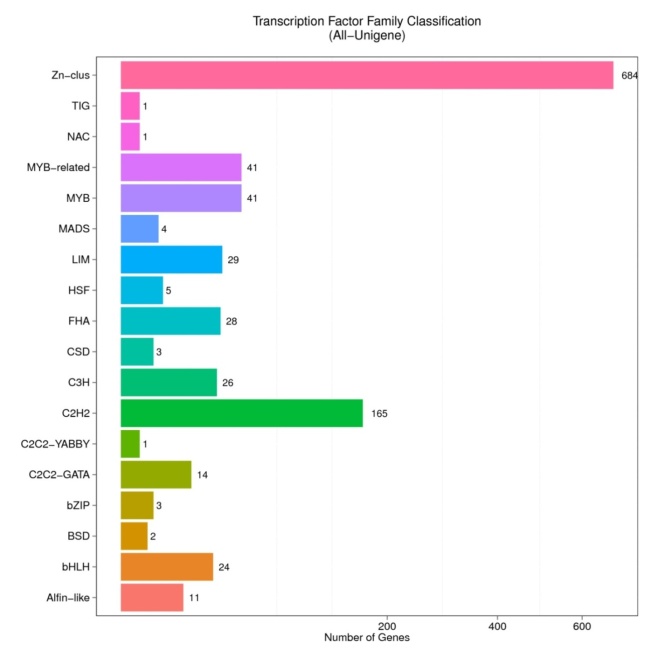


All

Down

Up

B

A
