# Supplementary material for: De novo transcriptomic assembly and mRNA expression patterns of Botryosphaeria dothidea infection with mycoviruses chrysovirus 1 (BdCV1) and partitivirus 1 (BdPV1)
Source: Virol J. 2018 Aug 13;15:126. doi: 10.1186/s12985-018-1033-4 (PMC6088430; doi:10.1186/s12985-018-1033-4)
Supplement: Supplementary file 11 — Figure S11. Gene Ontology (GO) enrichment terms analysis of DEGs in responsive to LW-P showing the main enriched processes related to (A) Biological process, (B) Cellular component, (C) Molecular function. (DOCX 715 kb) [file 12985_2018_1033_MOESM11_ESM.docx]

**Additional file 11: Figure S11**. Gene Ontology (GO) enrichment terms analysis of DEGs in responsive to LW-P showing the main enriched processes related to (A) Biological process, (B) Cellular component, (C) Molecular function.

A


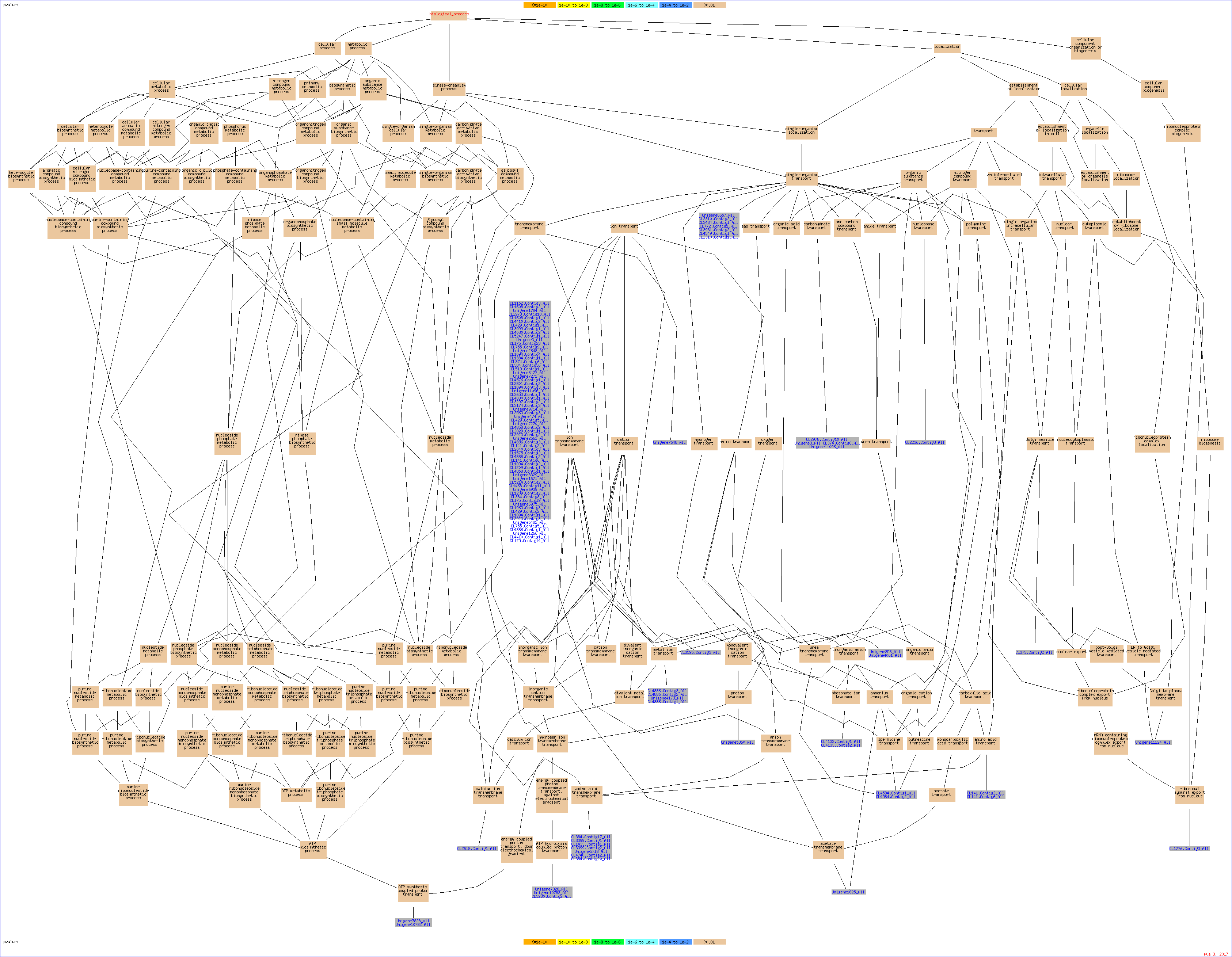


B


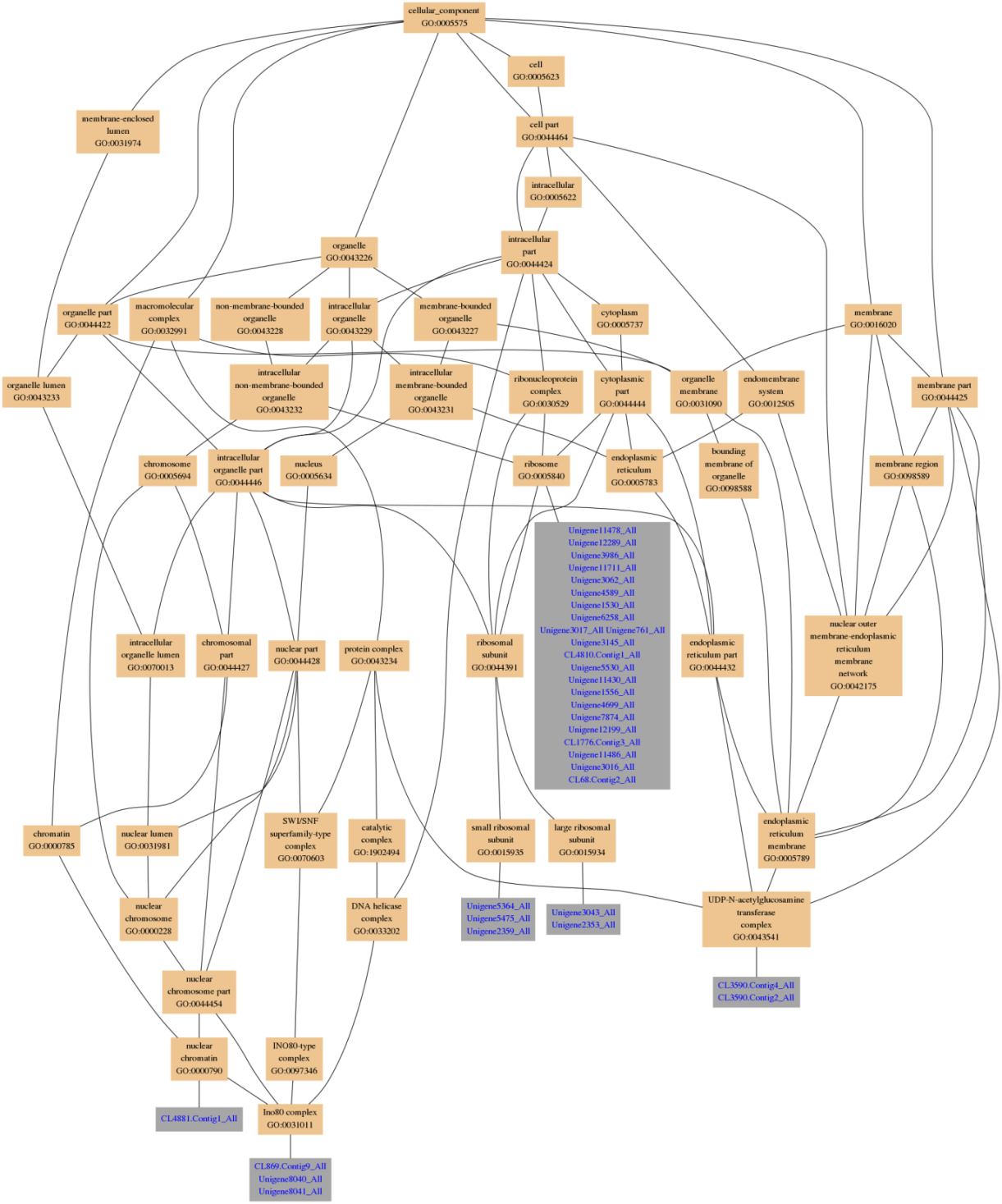


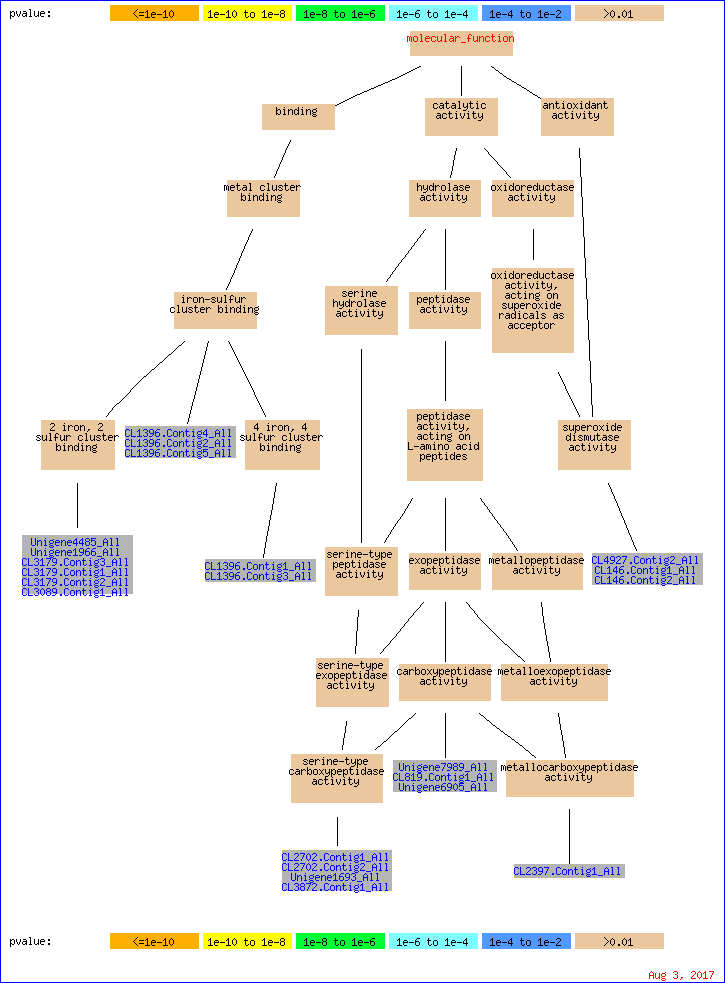


C
